# Matrilin3/TGFβ3 gelatin microparticles promote chondrogenesis, prevent hypertrophy, and induce paracrine release in MSC spheroid for disc regeneration

**DOI:** 10.1038/s41536-021-00160-0

**Published:** 2021-09-03

**Authors:** Alvin Bacero Bello, Yunkyung Kim, Sunghyun Park, Manjunatha S. Muttigi, Jiseong Kim, Hansoo Park, Soohong Lee

**Affiliations:** 1 School of Integrative Engineering, Chung-Ang University, Seoul, 06911 Korea; 2 Department of Medical Biotechnology, Dongguk University, Seoul, 04620 Korea; 3 Department of Life Science, CHA University, Seongnam, 13488 Korea

**Keywords:** Stem-cell differentiation, Biomaterials - cells, Mesenchymal stem cells, Regeneration

## Abstract

Degenerative disc disease (DDD) is the leading cause of excruciating lower back pain and disability in adults worldwide. Among the current treatments for DDD, cell-based therapies such as the injection of both disc- and non-disc-derived chondrocytes have shown significant improvements in the patients’ condition. However, further advancement of these therapies is required to not only ensure a supply of healthy chondrocytes but also to promote regeneration of the defective cells in the injury site. Here, we report that the incorporation of gelatin microparticles coloaded with transforming growth factor beta 3 and matrilin 3 promoted chondrogenic differentiation of adipose-derived mesenchymal stem cell spheroids while preventing hypertrophy and terminal differentiation of cells. Moreover, these composite spheroids induced the release of chondrogenic cytokines that, in turn, promoted regeneration of degenerative chondrocytes in vitro. Finally, injections of these composite spheroids in a rat model of intervertebral disc disease promoted restoration of the chondrogenic properties of the cells, thereby allowing regeneration of the chondrogenic tissue in vivo.

## Introduction

Intervertebral disks (IVDs) are fibro-cartilaginous tissues that lie between adjacent vertebrae of the spinal column. They are the key component of the spine that absorbs mechanical shock, imparts mobility, permits flexibility, and provides efficient support for the body^1,2^. These disks are primarily composed of a central nucleus pulposus (NP), concentric rings of annulus fibrosus (AF), and endplate, and they are rich in proteoglycans and various collagen proteins (collagen I and collagen II)^3^. However, aging, coupled with other contributing factors such as injury, smoking, diet, obesity, and extreme physical activity, inevitably causes progressive disintegration of IVDs, resulting in a condition commonly known as degenerative disc disease (DDD)^4–9^. DDD is the leading cause of excruciating lower back pain in adults worldwide and is often characterized by several morphological (bulging, herniation, and thinning) and molecular (tissue dehydration, cellular senescence, oxidative stress, and proteolytic damage) changes that ultimately lead to tissue collapse, structural failure, and loss of function^5,8,10^.

Treatment for DDD includes nonsurgical (occupational therapy, physical therapy, and medications like steroids, opiates, and nonsteroidal antiinflammatory drugs [NSAIDs]) and surgical interventions such as gene therapy, prosthetic total disc replacement (TDR), and cell-based therapy^11,12^. Cell-based therapies range from the use of disc-derived and non-disc-derived chondrocytes to treatments with stem cells such as the mesenchymal stem cells (MSCs)^13^. MSCs have long been used in tissue engineering and regenerative medicine for their ability to differentiate into different progenitor cells such as chondrocytes, osteocytes, and adipocytes. Many studies on DDD that attempted transplantation of MSCs and MSC-derived chondrocytes into the injury site have shown promising results and significant improvements in patients’ condition^14^.

Chondrogenic differentiation of MSCs in vitro has been attempted with both 2-D and 3-D cultures incubated in differentiation media supplemented with various chondrogenic induction molecules, such as transforming growth factor beta (TGF-β3) and fibroblast growth factors (FGFs)^15^. Although a 2D culture system is fast and easy, 3D cell aggregates called spheroids have been proven to be superior since the cells cultured in spheroids show increased expression of angiogenic, antiinflammatory, and chondrogenic markers. Moreover, these cells show improved survivability and thus offer greater regenerative capacity in transplantation procedures^16^. However, due to the 3D conformation of cell spheroids, the efficient transfer of nutrients, differential oxygen concentrations, and diffusivity of growth factors into the core have remained major concerns^16,17^. To address these issues, microcapsules in the form of hydrogel microparticles may be incorporated into the cell spheroids to deliver important proteins or supply oxygen in the core of the spheroids^18^. In our previous study, we showed that gelatin microparticles (GMPs) can be incorporated in adipose-derived MSC (ASC) spheroids without compromising cell viability^19^. GMPs are biocompatible and biofunctionalizable. Proteins such as TGF-β1 have been conjugated to GMPs to facilitate cartilage tissue regeneration^20^. Moreover, the physical properties of GMPs can also be modified for controlled and sequential release of TGF-β1 and BMP-2 for osteochondral tissue regeneration^21^.

In the present study, we generated GMPs conjugated with TGF-β3 and matrilin-3 and incorporated them into ASC cell spheroids. In comparison with the conventional chondrogenic inducer TGF-β1, TGF-β3 has been proven to show similar chondrogenic induction capacity but with enhanced cellular proliferation and survivability; thus, we utilized it in this experiment^22^. Matrilin-3, on the other hand, is an important ECM in cartilage tissues and has been proven to suppress hypertrophy of chondrocytes by binding to BMP-2 and thereby inhibiting the activity of the downstream hypertrophic gene *RUNX2*^23^. We hypothesize that the incorporation of TGF-β3- and matrilin-3-loaded GMPs in ASC cell spheroids (ASC–GMP–MATN3/TGF-β3) enhances the chondrogenic differentiation of ASCs via TGF-β3 while preventing hypertrophy and early terminal differentiation of cells via matrilin-3.

**Fig. 1:**
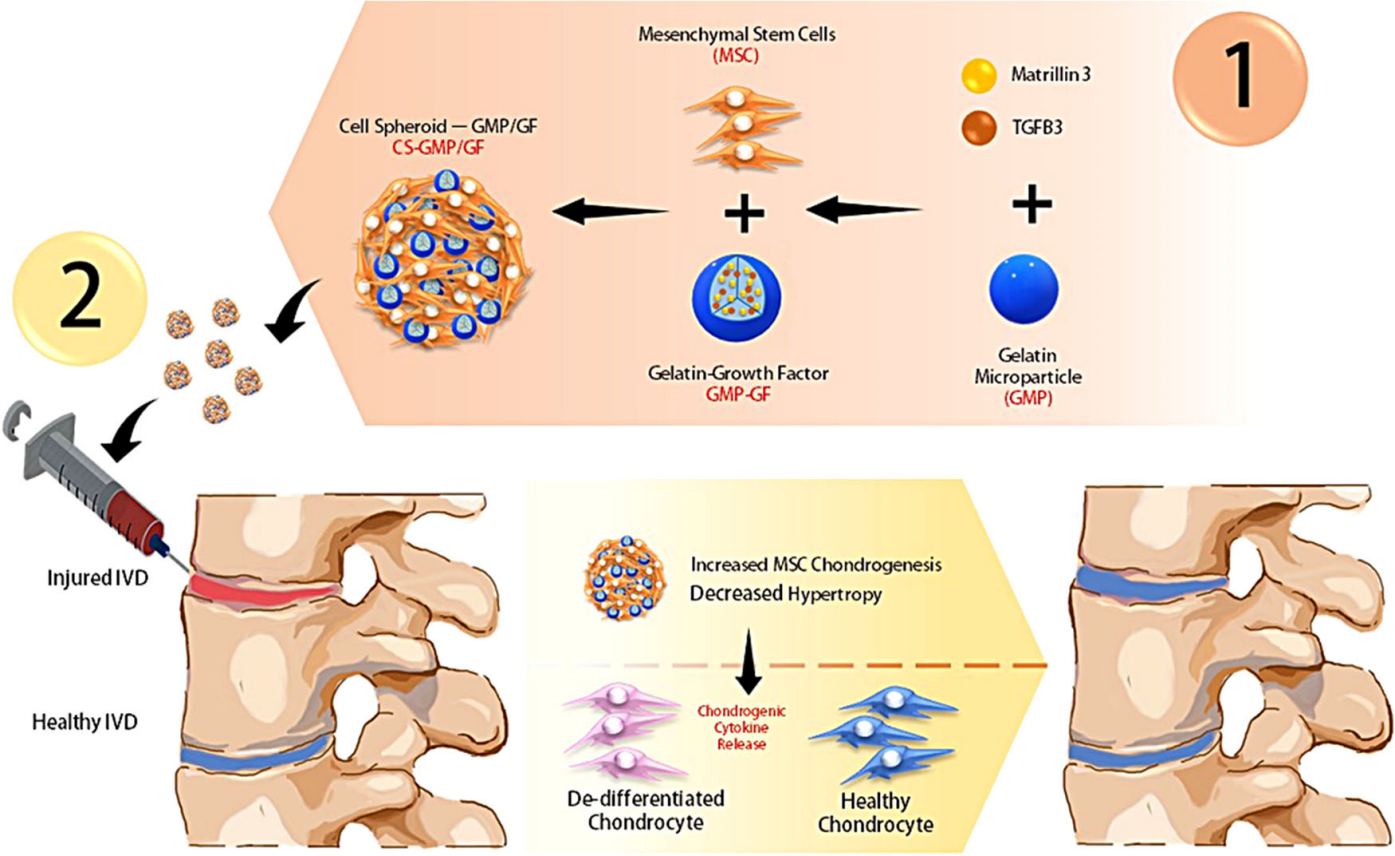
Research summary. Incorporation of TGFB3 and Matrilin-3-loaded gelatin microparticle (GMP) in ASC spheroids enhances chondrogenic differentiation of cells, inhibits hypertrophy, and promotes the secretion of chondrogenesis-enhancing cytokines that are essential for the regeneration of degenerated cells in intervertebral disc injuries.

We also sought to understand the effect of the ASC–GMP–MATN3/TGF-β3 spheroid composites on the regeneration of degenerated chondrocytes (DCs). With the enhanced chondrogenic differentiation of ASC–GMP–MATN3/TGF-β3 spheroids, we hypothesize that these spheroids secrete paracrine factors, including chondrogenesis-promoting cytokines such as TGF-β1, TGF-β2, and TGF-β3, and induce chondrogenic regeneration of dedifferentiated chondrocytes. Ultimately, we aimed to develop injectable GMP–ASC spheroids that could deliver new and healthy ASCs with enhanced chondrogenic properties that secrete cytokines to facilitate the regeneration of dedifferentiated chondrocytes as an alternative treatment for intervertebral disc diseases (Fig. 1).

## Results

### GMP microparticle incorporation did not affect proliferation and spheroid formation of ASCs

**Fig. 2:**
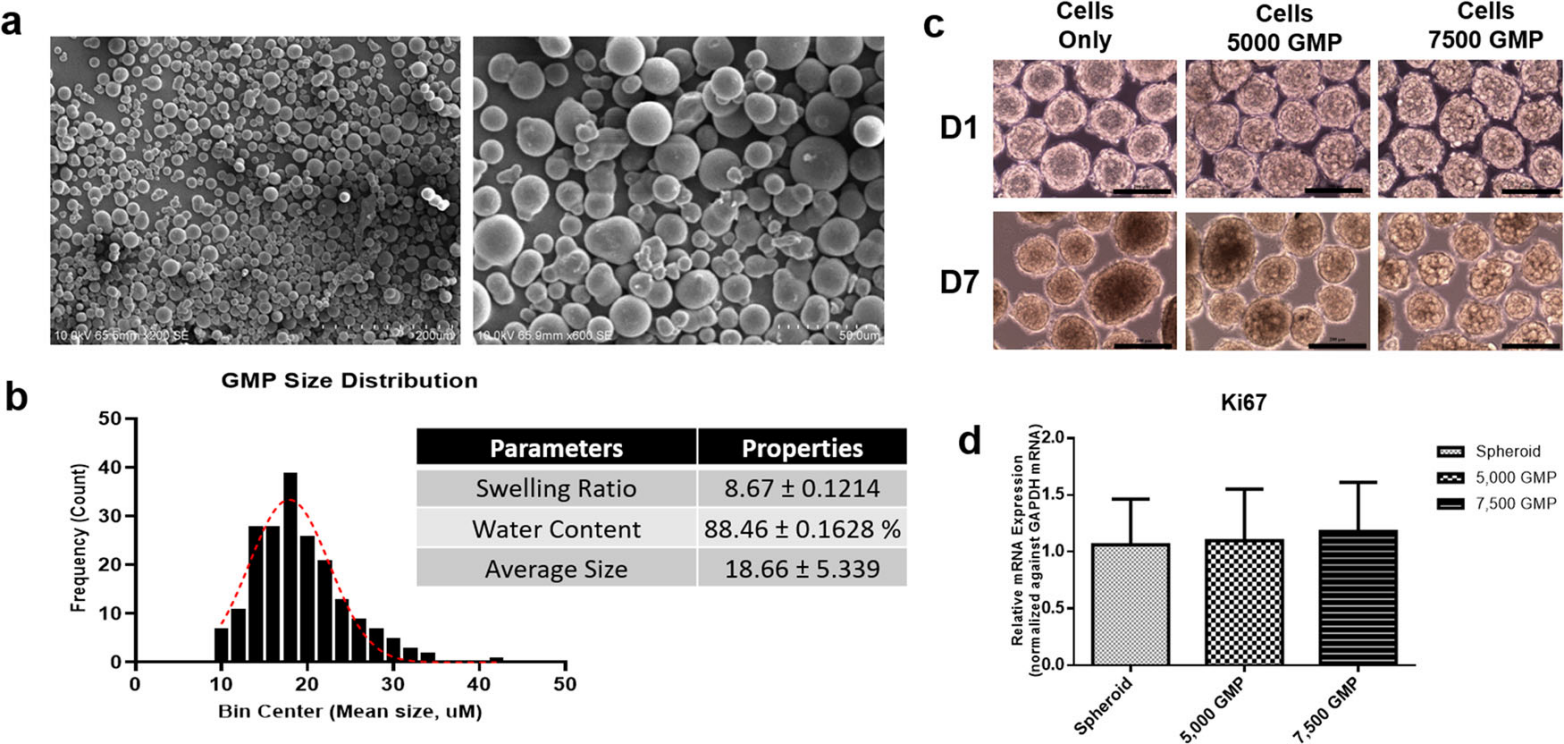
SEM images, physiochemical properties, and incorporation of gelatin microparticles in ASC spheroids. The GMPs are round, solid, and have a smooth surface with an average diameter of 18.66 ± 5.339 µm (**a, b**). The microparticles have a swelling ratio of 8.84 and water retention up to 85.20%. Incorporation of GMPs (5000 and 7500 GMPs) did not affect the proliferation and did not hinder the spheroid formation of ASCs, as also indicated by the expression of the proliferation marker Ki-67 (**c, d**; scale bars = 200 μm).

The GMPs (GMPs) were fabricated using the conventional water-in-oil emulsion method and were crosslinked with glutaraldehyde. Parameters such as gelatin type, concentration, crosslinking method, and length of incubation were previously optimized in the laboratory [19]. Our optimized protocol generates microparticles with less than 20 µm. The sizes of GMPs were maintained below 20 µm to achieve efficient incorporation of GMPs in ASC spheroids without compromising the ability of the cells to form intact aggregates. SEM and frequency-distribution analysis show that the particles were solid and round, had smooth surfaces, and had an average size of 18.66 ± 5.339 µm (Fig. 2). Moreover, the microparticles had a relative swelling ratio of 8.67 ± 0.1214 and water retention of up to 88.46 ± 0.1628%. GMP incorporation (up to 7.5 × 10^3^ GMPs per 5 × 10^5^ cells in each microwell) did not affect spheroid formation in ASCs, as indicated in brightfield images of the spheroids, which was verified by mRNA expression analysis of the proliferation marker Ki-67 (Fig. 2D).

### GMPs enable slow release of the growth factor TGF-β3 and the ECM protein matrilin-3

**Fig. 3:**
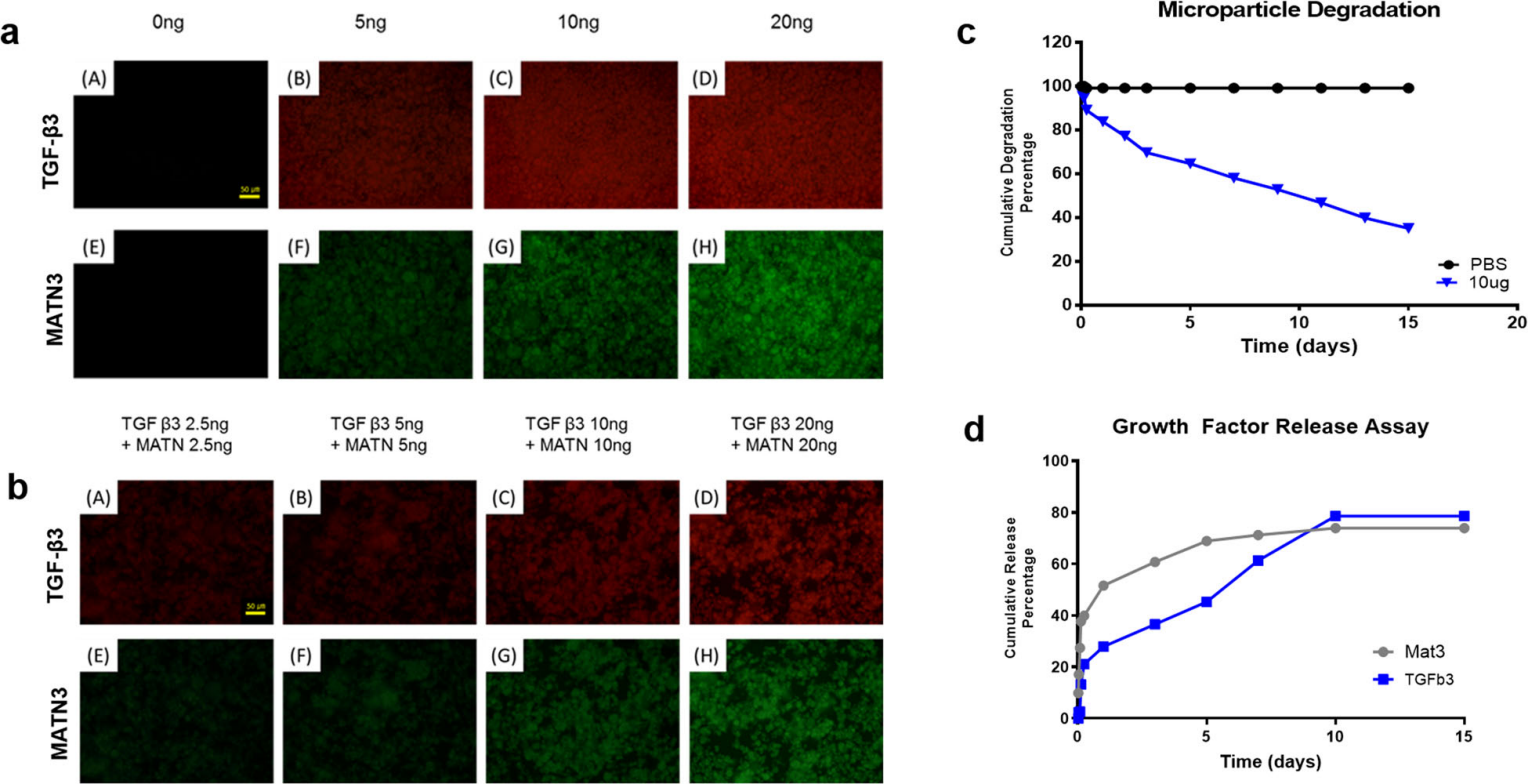
Conjugation and release of TGF-β3 and matrilin-3 from gelatin microparticles. Representative immunofluorescent images of TGF-β3- and MATN3-conjugated GMPs (**a, b**, scale bars = 50 µm). Degradation of GMP incubated in 10 µg/ml collagenase I in PBS measured via BCA protein assay (**c**). Cumulative release of 1 µg of TGF-β3 (activated form) and 1 µg of MATN from 1 mg of GMPs incubated in 10 µg/ml collagenase I (**d**).

To stably conjugate the growth factor TGF-β3 and the ECM protein matrilin-3 to the GMPs, conventional EDC/NHS conjugation was utilized. Covalent conjugation ensures slow and continuous release of the conjugated proteins in the microparticles. To verify the efficiency of conjugation, fluorophore-tagged monoclonal antibodies against TGF-β3 (Alexa Fluor 594) and Mat-3 (FITC) were utilized. As seen in Figs. 3A and 3B, higher concentrations of both TGF-β3 and MATN3 yielded higher fluorescent signals when separately conjugated to GMP. Moreover, coconjugation of both proteins in the GMP also showed consistent observations supporting secure immobilization of both proteins to GMPs. The degradation of GMPs was then assessed by incubating the GMPs in collagenase solution. In Fig. 3C, the total GMP concentration in the collagenase solution reduced by 50% after 10 days. In contrast, incubation in PBS did not degrade the GMPs, and the degradation rates increased when GMPs were incubated in higher concentrations of collagenase solution (data not shown). In theory, the covalent conjugation of growth factors to the gelatin involves the formation of amide bonds between the carboxyl moiety of gelatin and the primary amine of the growth factor. Amide bonds are relatively stable but could be broken down by hydrolysis via a strong base or acid. However, in this experiment, the release of the growth factor is dependent on the degradation of the gelatin microparticles. Gelatin is highly susceptible to degradation by several matrix metalloproteinases (MMPs), such as MMP 2, and MPP9 (gelatinases, collagenases) secreted by the cells that degrade the gelatin into small fragments. Upon degradation, the fragments containing the growth factor are then released to the solution and the growth factors can now freely bind to its receptor on the surface of the cells. The diffusion and availability of the growth factors is not entirely dependent on the breakage of amide bond but by the degradation of gelatin into small, diffusible fragments releasing functional growth factors. To verify this, we performed a sandwich ELISA against MATN3 and TGF-β3. Both proteins showed burst release during the first few hours of incubation with a subsequent slow release that was still present 15 days post incubation (Fig. 3D). These results suggest that covalent conjugation to GMPs enables the slow and consistent release of the growth factors, which is useful for long-term cell cultures such as cell differentiation.

### Composite GMP microparticles induced chondrogenic differentiation of ASC spheroids

In our previous study on the effect of matrilin-3 on the chondrogenic differentiation of ASCs, we found that 10 ng/mL of matrilin-3 showed the highest expression of chondrogenic markers and lower expression of hypertrophy markers and thus opted to use the same concentration for this study^24^. Similarly, for TGF-β3, we adapted the concentration of TGF-β3 in conventional chondrogenic medium, which is 10 ng/mL. Since all the proteins will come from and will be released from the GMPs for the whole duration of differentiation, we decided to conjugate 100 ng TGF-β3 (GMP-TGF-β3), 100 ng MATN3 (GMP-MATN3), or 100 ng of TGF-β3 and MATN3 (GMP–MIX) per 7,500 GMPs and incorporate them into 5.0 × 10^5^ ASCs.

**Fig. 4:**
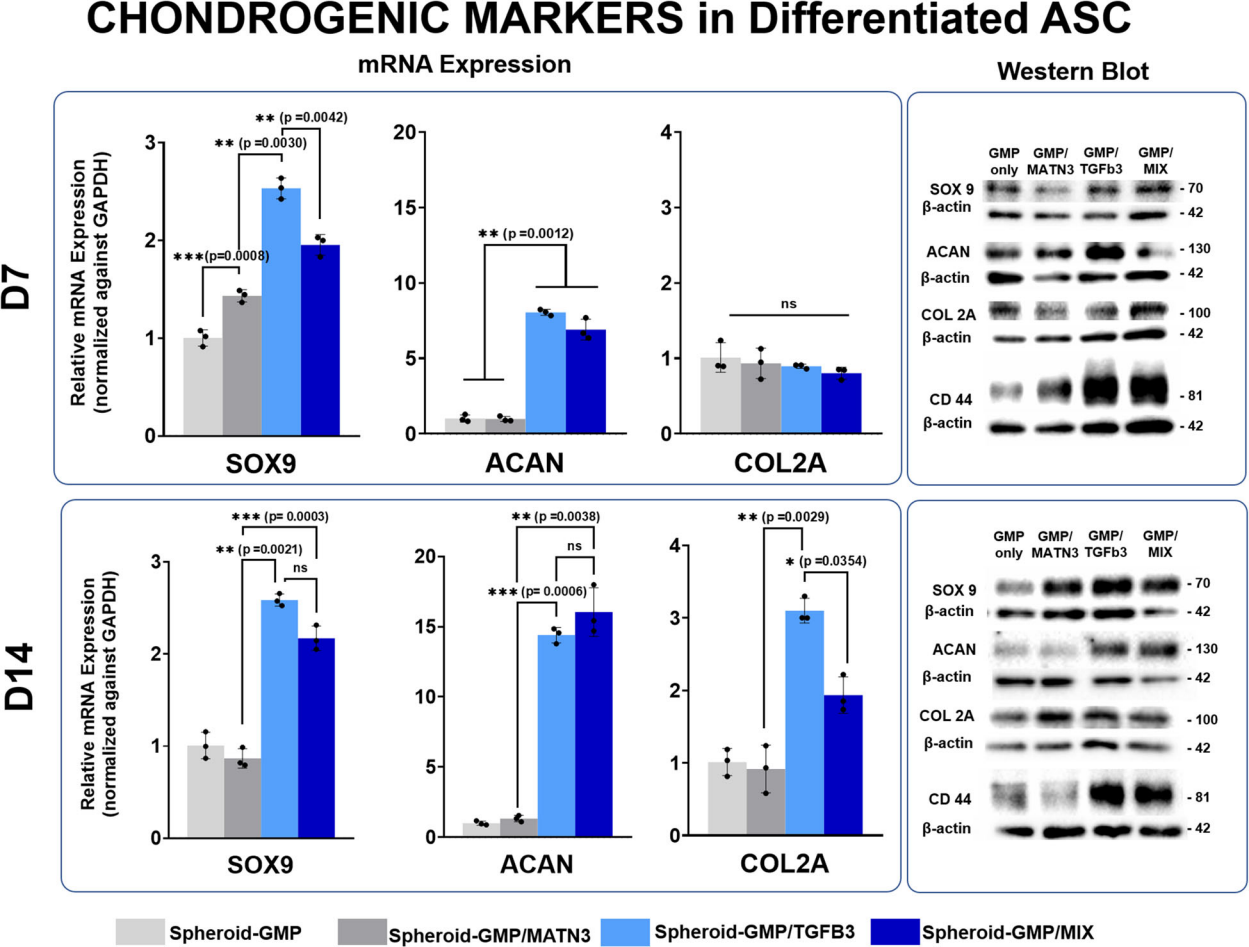
Time-dependent chondrogenic differentiation of ASC spheroids. Quantitative real-time gene expression analysis and immunoblot analysis of the chondrogenic markers SOX9, ACAN, COL2A, and CD44; 7 days (top panel) and 14 days (bottom panel) post differentiation. qRT PCR data were normalized against GAPDH expression, while western blot data were normalized against B-actin control. The data are representative of three independent experiments, each experiment done in triplicate. Error bars denote the means ± standard error of mean (sem) (ns = not significant, *p < 0.05, **p < 0.01, ***p < 0.001; ****p < 0.0001). Individual data point and *p*-values for significance were indicated in the graph.

To assess the degree of differentiation of the composite spheroids, RNA and protein samples were collected at 7 and 14 days post differentiation and subjected to qRT-PCR and western blot analyses (Fig. 4). Differential gene expression analyses showed that, among all the setups, the spheroids containing TGF-β3 showed the highest expression of the chondrogenic markers SOX9 (2.53 ± 0.106-fold, Day 7; 2.59 ± 0.067-fold, Day 14) and ACAN (8.048 ± 0.201-fold), followed by the GMP–MIX group with SOX9 (1.95 ± 0.106-fold, Day 7; 2.170 ± 0.133-fold, Day 14) and ACAN (6.91 ± 0.698-fold, Day 7; 16.034 ± 1.728, Day 14). Interestingly, the expression of collagen type 2 A was not apparent on Day 7 but showed a significant increase at Day 14 in both the GMP–TGF-β3 group (3.01 ± 0.171-fold) and GMP–MIX group (1.936 ± 0.251-fold). The increase in magnitude in the expression of these gene markers between days 7 and 14 suggests that the conjugated chondrogenic factors were consistently being released from the microparticles. In contrast, both groups containing MATN3 (i.e., GMP–MATN3 and GMP–MIX) showed lower chondrogenic differentiation compared with GMP–TGF-β3 alone. Initially, we thought that MATN3 negatively regulates chondrogenic differentiation; however, the expression of chondrogenic markers in the MATN3 group was not significantly lower than that with the unconjugated GMP. Moreover, the expression of SOX9 was significantly higher in the MAT3 group (1.432 ± 0.062-fold) compared with unconjugated GMP at day 7 of differentiation. We then hypothesized that MATN3 may not have a direct effect on the chondrogenesis of ASC spheroids but may be important in the terminal differentiation and subsequent hypertrophy of differentiating chondrocytes.

**Fig. 5:**
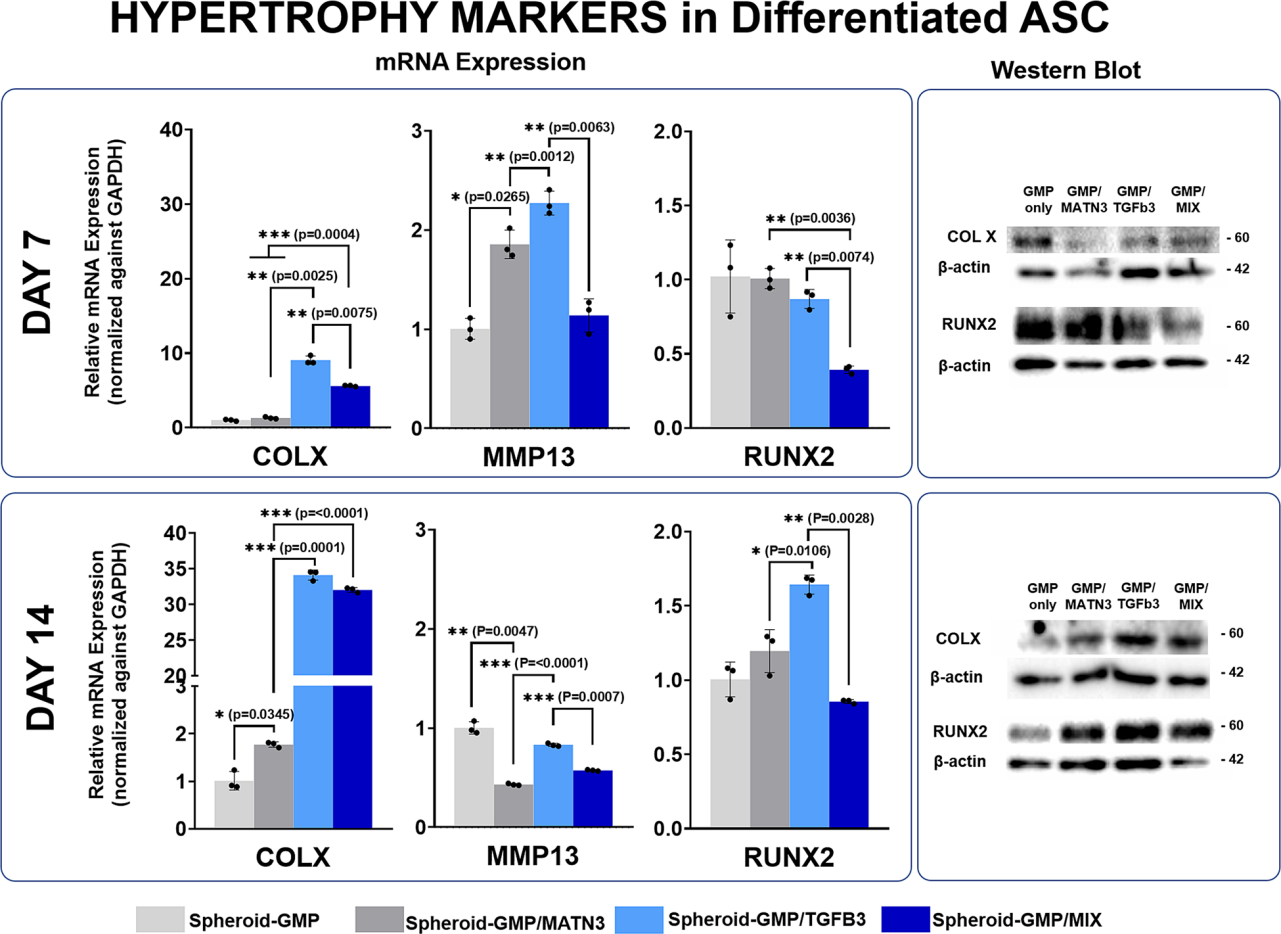
Time-dependent expression of hypertrophy markers in ASC spheroids. Quantitative real-time gene expression analysis and immunoblot analysis of the hypertrophy markers COLX, MMP13, and RUNX2; seven days (top panel) and 14 days (bottom panel) post differentiation. qRT-PCR data were normalized against GAPDH expression while western blot data were normalized against B-actin control. The data are representative of three independent experiments, each experiment done in triplicate. Error bars denote the means ± standard error of mean (sem) (ns = not significant, *p < 0.05, **p < 0.01, ***p < 0.001; ****p < 0.0001). Individual data point and *p*-values for significance were indicated in the graph.

To investigate the possible effect of matrilin-3 on the terminal differentiation of ASCs into chondrocytes and the hypertrophy of cells, we evaluated the expression of various markers of chondrocyte hypertrophy, such as COLX, RUNX2, and the ECM protein MMP13 (Fig. 5). The GMP–MIX group showed a remarkable reduction in the expression of RUNX2 (0.392 ± 0.025-fold, Day 7; 0.855 ± 0.013-fold, Day 14) and MMP13 (1.139 ± 0.167-fold, Day 7; 0.574 ± 0.007-fold, Day 14) compared to that in the GMP-TGF-β3 group (0.870 ± 0.064 -fold, Day 7; 1.643 ± 0.064-fold, Day 14). Moreover, the hypertrophy marker COLX 10 was significantly reduced in the GMP–MATN3 group compared to that in the GMP–TGF-β3 group (almost 7-fold and 20-fold at days 7 and 20, respectively). These results suggest the possible function of MATN3 in inhibiting the expression of hypertrophy markers, since the groups containing MATN3 (i.e., GMP–MATN3 and GMP–MIX) showed reduction of hypertrophic markers. Overall, based on gene and protein expression analyses, incorporation of TGF-β3- and MATN3-conjugated microparticles in ASC spheroids effectively induced the chondrogenic differentiation of cells while preventing hypertrophy and terminal differentiation of chondrocytes.

**Fig. 6:**
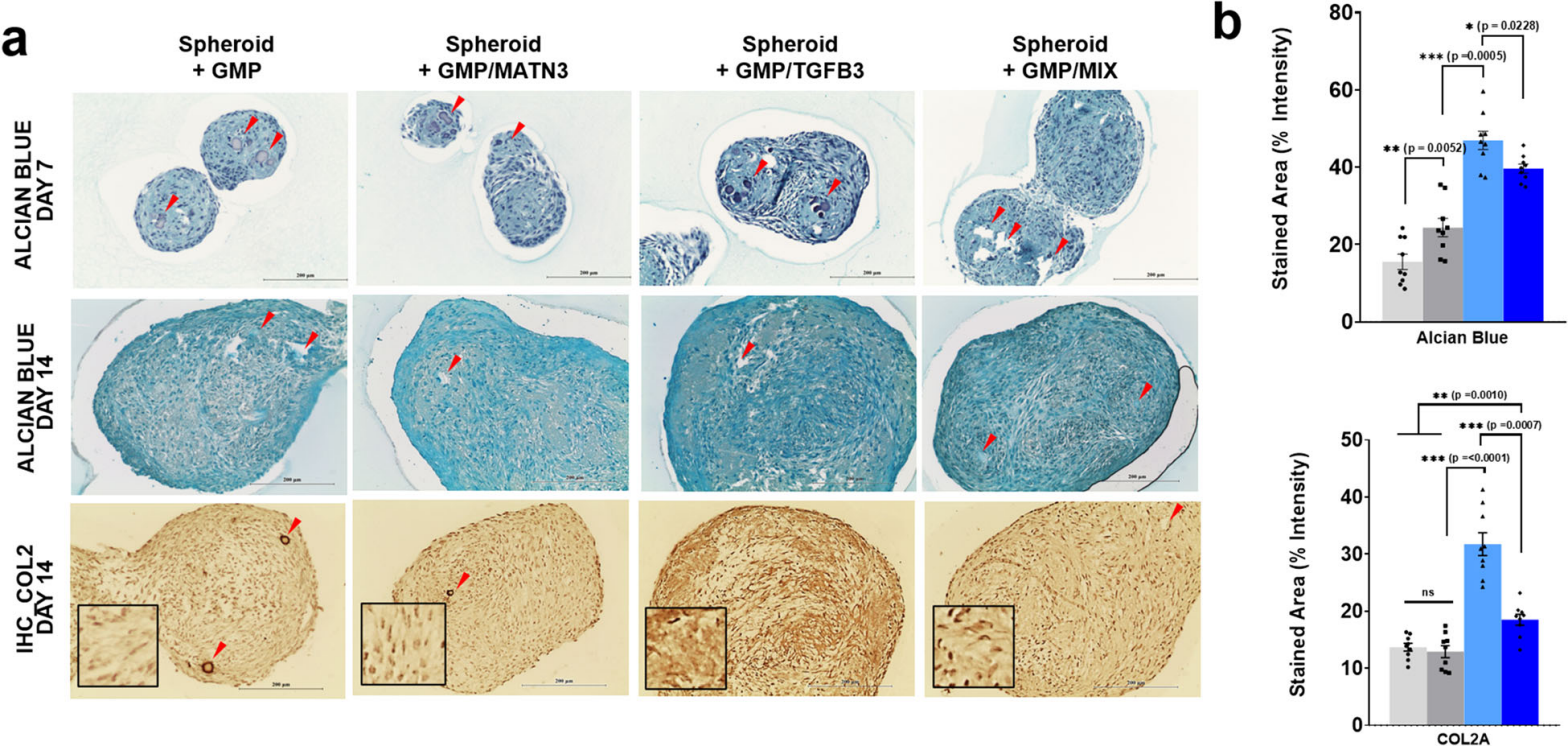
Alcian blue staining and immunohistochemical analysis of collagen 2 A in composite spheroids (Day 7 and 14). Representative bright-field images of Alcian blue staining seven days post differentiation (**a**, topmost row), 14 days post differentiation (**a**, middle row), and immunohistochemical labeling of collagen 2A on 5-µm sections of composite spheroids (**a**, bottom row). Scale bars = 200 µm. Color and staining intensities of each section were digitally quantified using ImageJ software (**b**). The images are representative images of 9 sections from three independent spheroids. Red arrows indicate the location of gelatin microparticles in the differentiating ASC spheroids.

Chondrogenic differentiation of ASC spheroids results in the development of cartilage and ECM that are rich in proteoglycans, such as aggrecans and various collagen proteins. To further assess the chondrogenic differentiation of ASCs, spheroids were collected, fixed, sectioned, and analyzed via Alcian blue staining of aggrecan and collagen type 2 A immunostaining (Fig. 6). Among all the groups, GMP–TGF-β3 and GMP–MIX showed the highest intensity of staining, followed by GMP–MATN3, for both day-7 and day-14 samples. These results support the findings of both mRNA and western blot gene expression analyses. Similar to Alcian blue staining, collagen type II was heavily stained in GMP–TGF-β3, followed by GMP–MIX, GMP–MATN3, and the GMP groups. These results support the trend we observed with the gene and protein analyses of the differentiated ASC spheroids.

### Differentiated ASC spheroids containing composite GMP microparticles secrete chondrogenic cytokines

**Fig. 7:**
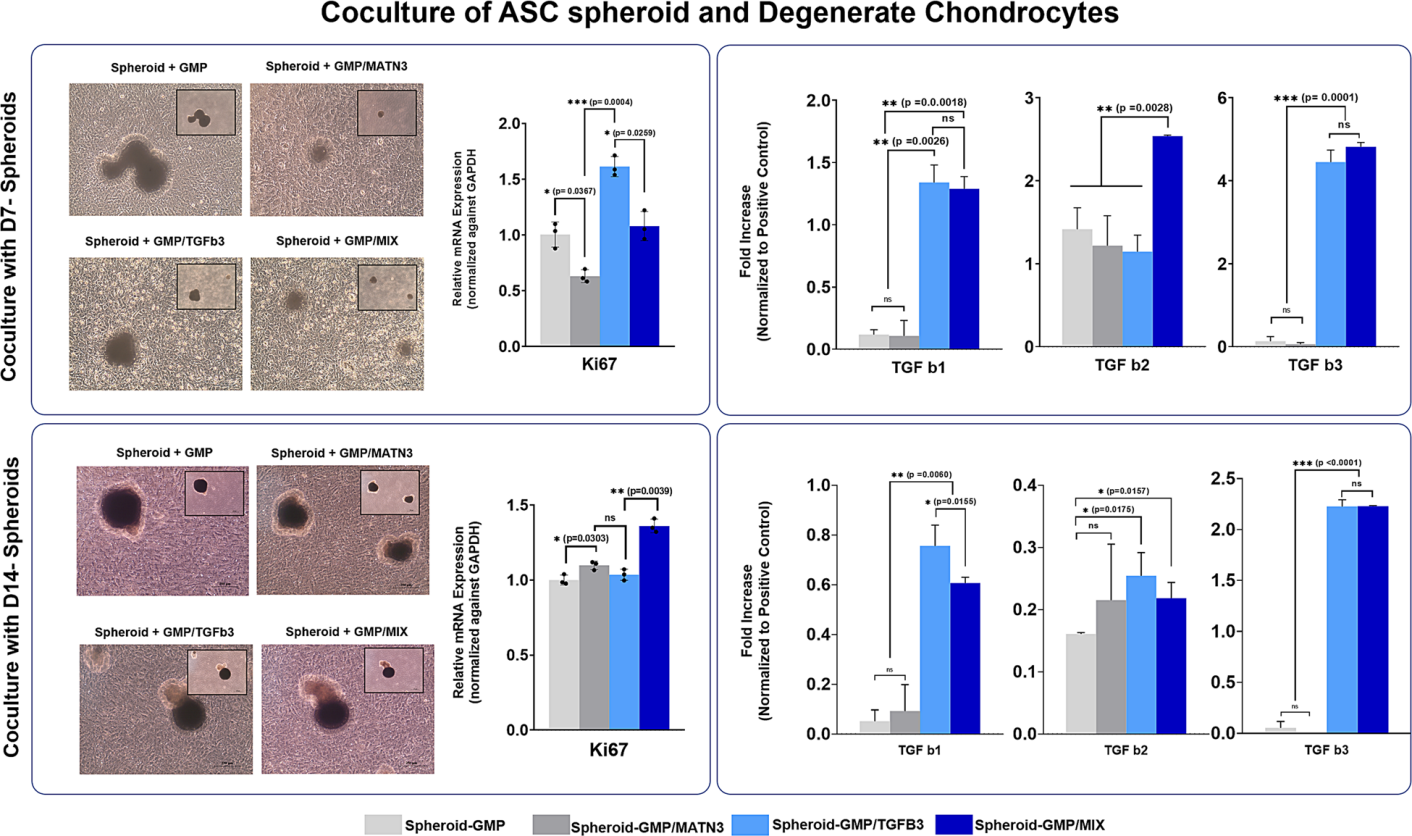
Coculture of D7- and D14-differentiated hASC spheroids with degenerated chondrocytes. Representative bright-field images of the cocultures of D7-differentiated ASC spheroids (top panel) and D14 differentiated spheroids (bottom panel) with degenerated chondrocytes (scale bars = 200 µm). Quantitative real-time gene expression analysis proliferation marker Ki-67. Quantitative analysis of secreted TGF-β isoforms from hASC spheroids (right panel). RT-PCR data were normalized against GAPDH expression. The data are representative of three independent experiments in triplicate. Error bars denote the means ± standard error of mean (sem) (ns = not significant, *p < 0.05, **p < 0.01, ***p < 0.001; ****p < 0.0001). Individual data point and *p*-values for significance are indicated in the graph.

We hypothesize that upon differentiation of ASC spheroids into chondrocytes, the spheroids then secrete chondrogenic cytokines that support the survival and dedifferentiation of DCs. To assess this, we performed a coculture experiment between the 7- and 14-day differentiated spheroids with DCs (Fig. 7). In this study, the DCs were collected from informed patients, and were subcultured up to passage 6 to ensure late-stage differentiation. Cells at passage 6 were used for the coculture study, while the chondrogenic and hypertrophic properties of the DCs were analyzed and compared with a fresh and healthy batch of chondrocytes also collected from the same patient (Supplementary Fig. 3).

First, we analyzed whether the spheroids are indeed secreting chondrogenic cytokines via a cytokine array. Out of 30 cytokines, the secretion of transforming growth factor beta family of proteins (TGF-β1, TGF-β2, and TGF-β3) was increased in both GMP–TGF-β3 and GMP–MIX both from 7-day-and 14-day-differentiated ASC spheroids (Fig. 7). Upon further analysis, cytokines such as interleukin-6 (IL-6) and some matrix metalloproteinases (MMPs) and tissue inhibitor of metalloproteinases (TIMPs) were also differentially secreted among the groups. IL-6 is a pleiotropic cytokine (acts as both a pro- and anti-inflammatory agent) essential for immune regulation and tissue regeneration (Supplementary Figs. 4 and 5). It was previously determined to be upregulated and an important secretory factor during chondrogenic and osteogenic differentiation of MSCs^25,26^.

At Day 7, the levels of IL-6 secretion in GMP-TGF and GMP-MIX were slightly less than those in the GMP and GMP-MATN3 groups. However, with further differentiation (i.e., day-14 spheroids), IL-6 secretions were significantly higher in the GMP–TGF and GMP–MIX groups compared to those in the GMP and GMP–MATN3 groups. Consistent with our mRNA and protein analyses, IL-6 secretion was lower in GMP–MIX than in GMP–TGF-β3 at day 14, supporting the role of MATN3 in suppressing the hypertrophy of ASCs.

On the other hand, MMPs and TIMPs are essential for the differentiation of cells and formation of the ECM. MMPs are secreted proteolytic enzymes that degrade the ECM and are regulated by their specific TIMPs^27^. In the context of cartilage tissues, the balance between MMPs and their inhibitors determines the severity of osteo- and rheumatoid arthritis^28^. Consistent with our findings for IL-6 secretion, TIMP1 and TIMP2 were decreased at Day 7 in GMP–TGF and GMP-MIX groups but were increased at Day 14 compared with GMP and GMP–MATN3 groups as the spheroids underwent further differentiation.

### Differentiated ASC spheroids containing composite GMP microparticles promote regeneration of degenerated chondrocytes

Upon confirming the secretory profiles of the chondrogenic spheroids, we then sought to analyze whether these secretions would promote restoration of the chondrogenic properties of DCs. To assess this, we performed a coculture experiment using the composite spheroids and DCs in chondrogenic medium without further addition of other chondrogenic growth factors. We hypothesized that the secretions from the composite spheroids would provide the necessary paracrine factors and morphogens for regeneration of cells. The coculture of DCs with composite spheroids did not affect the proliferative capacity of DCs (Fig. 7). Interestingly, GMP–TGF-β3 and GMP–MIX showed a slight increase in the expression of Ki-67 both in Day-7 and Day-14 spheroid coculture setups, which suggests that ASC spheroids might have also induced better proliferation of DCs.

**Fig. 8:**
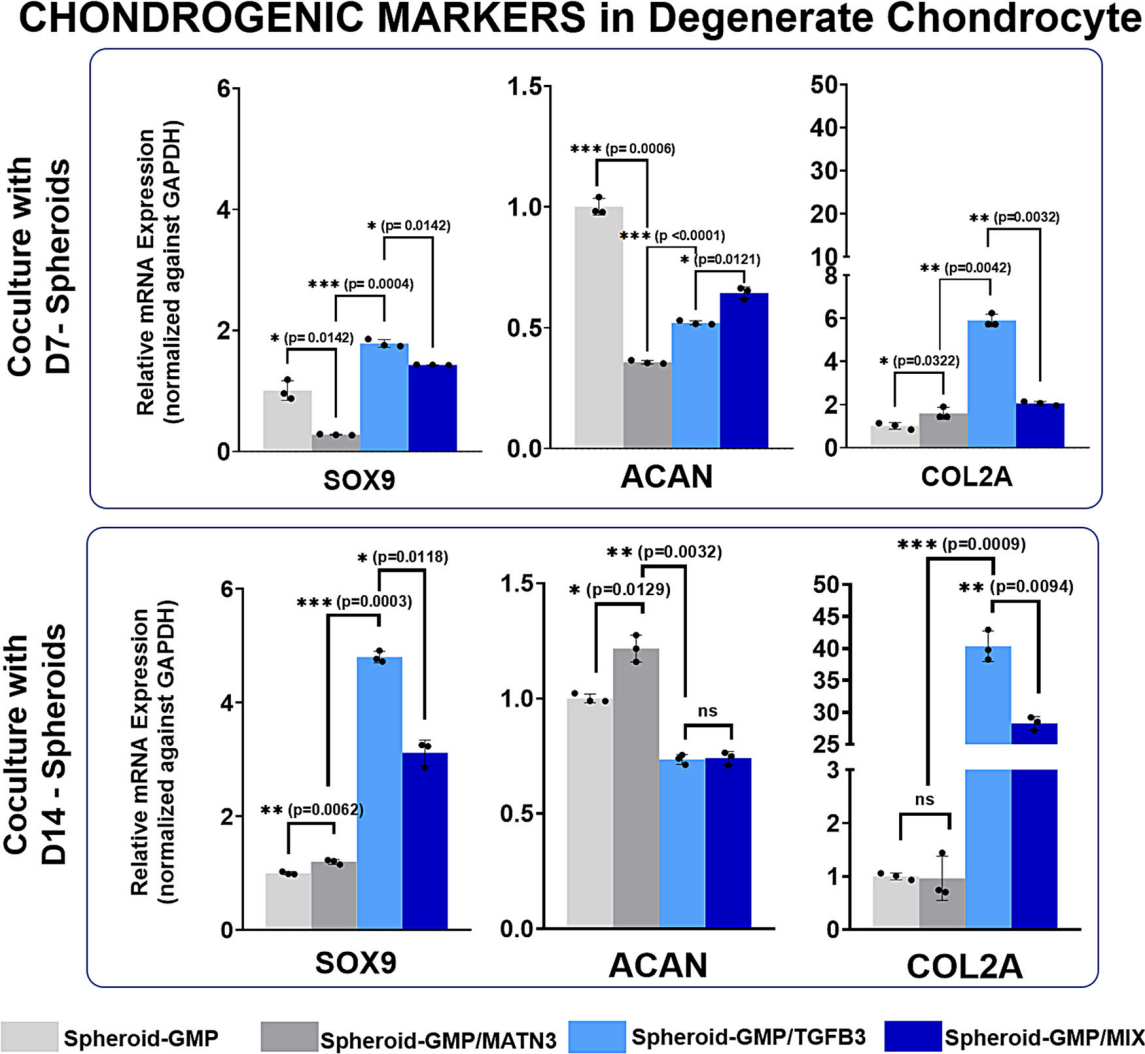
Expression of chondrogenic markers in degenerated chondrocytes cocultured with D7 and D14 spheroids. Quantitative real-time gene expression analysis of the chondrogenic markers SOX9, ACAN, and COL2 in degenerated chondrocytes cocultured with D7 and D14 spheroids. RT-PCR data were normalized against GAPDH expression. The data are representative of three independent experiments in triplicate. Error bars denote the means ± standard error of mean (sem) (ns = not significant, *p < 0.05, **p < 0.01, ***p < 0.001; ****p < 0.0001). Individual data points and *p*-values for significance are indicated in the graph.

After seven days of coculture, the chondrogenic markers SOX9 and Col2A were increased in DC-cocultured GMP–TGF-β3 and GMP–MIX composite spheroids in both Day-7 and Day-14 spheroid setups (Fig. 8). In contrast to what we expected, the expressions of ACAN in the GMP–TGF-β3 and GMP–MIX were lower than those in the GMP and GMP–MATN3 for both D7 and D14 spheroid setups. These findings suggested that the expression of ACAN over time in DCs has a different molecular mechanism compared with the chondrogenic differentiation of ASCs. Moreover, some scientists suggest that caution must be observed while using ACAN, since its expression over time shows some inconsistencies and thus may not always be a good indicator of chondrogenesis, especially in DCs^29^. These findings also suggested that the expression of SOX9 is sufficient to regain the cell’s response to all chondrogenic stimuli and, thus, its expression alone can indicate the chondrogenic capacity of cells^30^.

**Fig. 9:**
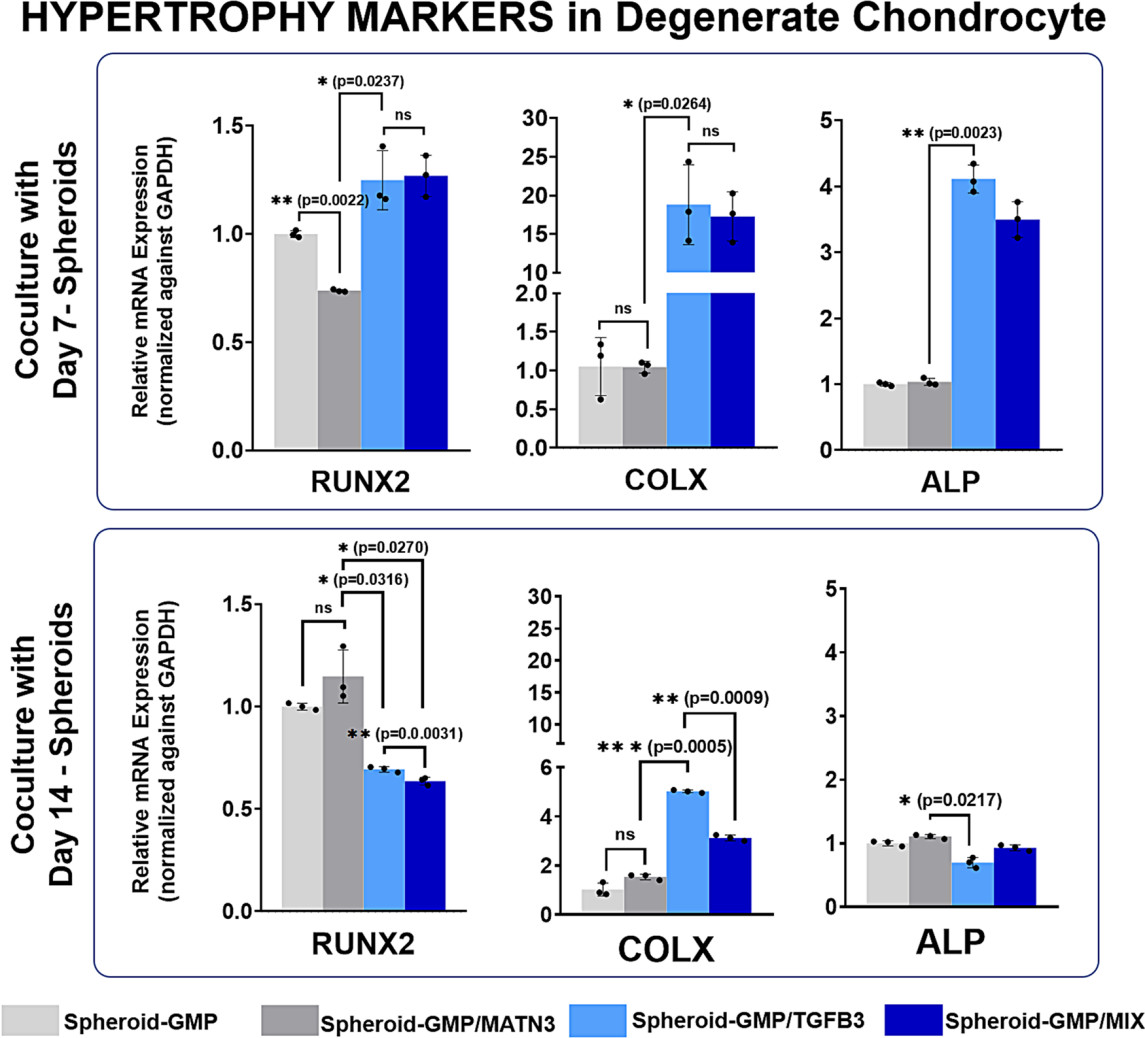
Expression of hypertrophy markers in degenerated chondrocytes cocultured with D7 and D14 spheroids. Quantitative real-time gene expression analysis of the hypertrophy markers RUNX2, COLX, and ALP in degenerated chondrocytes cocultured with D7 and D14 spheroids. RT-PCR data were normalized against GAPDH expression. The data are representative of three independent experiments in triplicate. Error bars denote the means ± standard error of mean (sem) (ns = not significant, *p < 0.05, **p < 0.01, ***p < 0.001; ****p < 0.0001). Individual data points and p-values for significance are indicated in the graph.

Finally, we assessed the expression of different hypertrophic markers in the cocultured DCs (Fig. 9). The expression levels of the hypertrophy markers COLX, RUNX2, and ALP were lower compared to those in GMP–TGF-β3 and GMP–MIX. Interestingly, for both D7 and D14 spheroid setups, the expression of hypertrophic markers in GMP–MIX is lower than that in GMP–TGF-β3, supporting the possible role of MATN3 in suppressing hypertrophy.

### Differentiated ASC spheroids promote regeneration of chondrogenic properties in a rat model of intervertebral disc injury

**Fig. 10:**
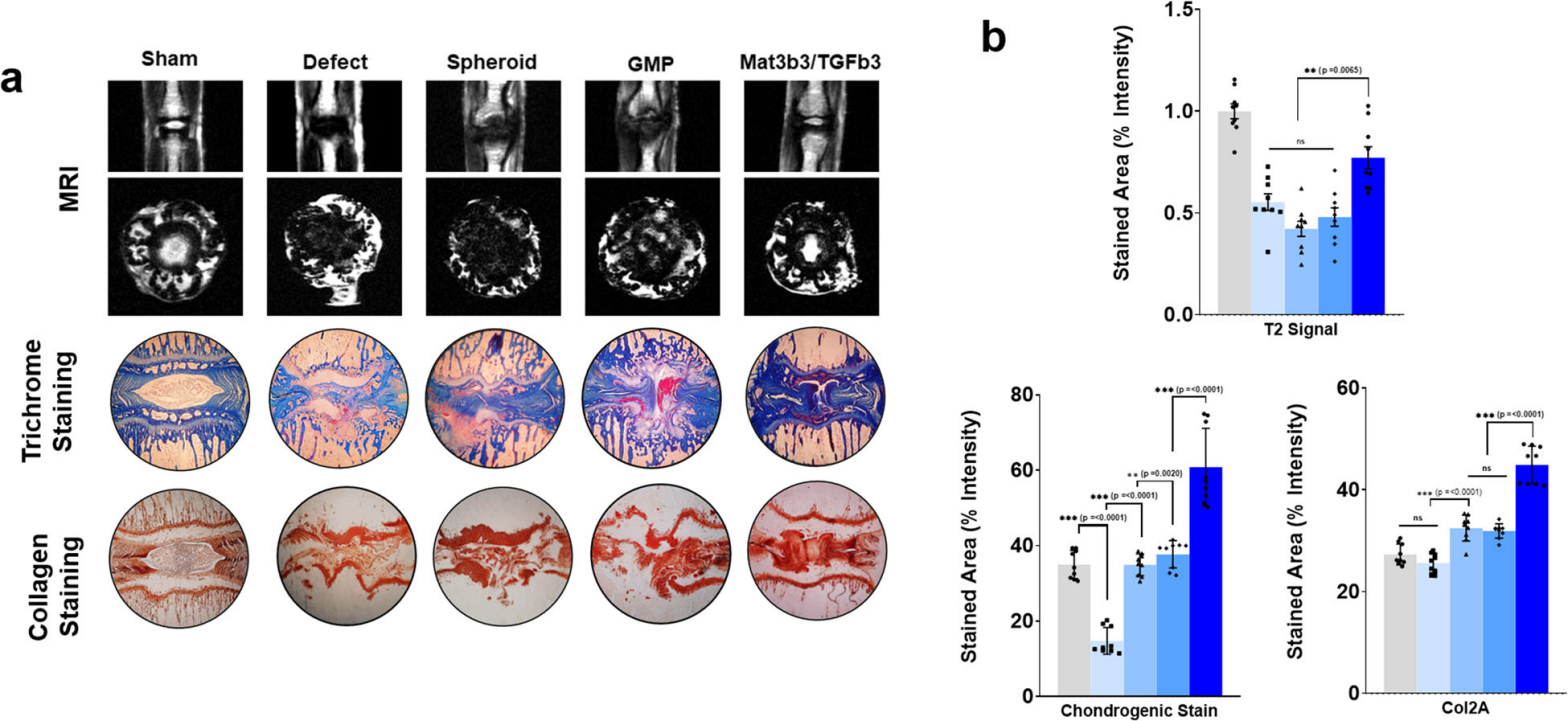
Radiographic and gross histological staining of rat intervertebral disks. Lateral MRI scans of IVD segments indicating the presence of cartilaginous tissue between adjacent rat-tail vertebrae (**a**, top panel). Representative images of the trichrome staining on 10-µm IVD sections (**a**, middle panel). Representative images of collagen type-2 immunostaining on 10-µm IVD tissue sections (**a**, bottom panel). The images are representative sections from nine independent IVD sites from three mice (3 IVD sites/mice). Quantitative digital analyses of the MRI scans, trichome staining, and Collagen type-2 immunostaining (**b**). Error bars denote the means ± standard error of mean (sem) (ns = not significant, *p < 0.05, **p < 0.01, ***p < 0.001; ****p < 0.0001). Individual data points and *p*-values for significance are indicated in the graph.

In order to determine the regenerative capacity of the GMP spheroids, we induced intervertebral disc degeneration by performing a mouse tail injury model. In this experiment, the intervertebral disks of the mouse tail were punctured subcutaneously with a needle to induce tissue damage and degeneration. A week after the induction of disc degeneration, composite spheroids were then implanted at the injury site replacing damage tissue and inducing regeneration in the injury site. Six (6) weeks post transplantation of the composite spheroids, the rats were sacrificed, and the tail was subjected to MRI and histological staining. Lateral and cross-sectional radiographs of the IVD show higher signal intensity of the NP in GMPs containing both MATN3 and TGF-β3 compared with all other groups. Trichome staining and immunostaining of the tissue sections also showed the greatest regeneration in GMPs containing both proteins, as indicated by the intensity of collagen signal present at the center of the IVD (Fig. 10). This supports the regenerative capacity of composite spheroids in the rat tail IVD model.

## Discussion

The current therapies for DDD include both nonsurgical and surgical techniques. Nonsurgical methods such as physical therapy, occupational therapy, and the use of nonsteroidal antiinflammatory drugs address the problem by temporarily alleviating the pain, while surgical techniques such as gene therapy, total disc replacement, and cell-based therapies work primarily by replenishing chondrogenic cells or completely replacing the damaged tissue.

Among cell-based therapies, the use and injection of chondrogenically differentiated mesenchymal stem cells have been popular for the following reasons: (1) MSCs are widely characterized, (2) MSC sources are abundant and readily accessible, (3) differentiation has been previously established, (4) and the process is easy, cheap, and proven to be replicable^31^. To promote chondrogenesis of MSCs, consistent addition of several growth factors such as various TGF-β proteins and FGF in the culture medium is required. However, it is important to note that the sequence of events in MSC chondrogenesis culminates with terminal differentiation, leading to hypertrophy of cells^32^. Chondrocyte hypertrophy is undesirable in the context of cartilage tissue regeneration since this process is normally accompanied by apoptosis, abnormal formation, and calcification of the ECM components^33^. The use of hypertrophic chondrocytes in DDD may induce other problems and further complications, leading to graft failure and unsuccessful tissue regeneration.

In this study, we found that incorporation of GMPs neither hindered the formation of ASC spheroids nor affected the proliferation of cells. Covalent conjugation of GMPs with the growth factor TGF-β3 and the ECM protein MATN3, on the other hand, led to the slow and steady release of proteins from the GMPs. This property was validated as the cells continuously underwent chondrogenesis, even after 14 days from the initial incorporation of the growth factor-loaded GMPs. Moreover, mRNA and immunoblot assays showed that the incorporation of spheroids with GMPs loaded with the growth factor TGF-β3 and the ECM protein MATN3 enhanced the chondrogenic differentiation of ASC spheroids while preventing terminal differentiation and hypertrophy of cells. These composite spheroids released cytokines necessary for chondrogenesis and thus led to the regeneration of the chondrogenic properties of DCs in coculture experiments. Finally, in vivo injection of the composite spheroids led to increased chondrogenesis in IVD and COL2A deposition at the injury site in a rat model of DDD.

There have been many attempts on utilizing chondrogenically differentiated MSCs in conjunction with biomaterials for the treatment of DDD. For instance, bone marrow-derived MSCs (BM-MSCs) are normally embedded in hydrogels such as atelocollagen, fibrin, alginate, and hyaluronic acid, to improve cell survivability, chondrogenic differentiation, and to ensure transplantability of differentiated MSCs^34–37^. Although these techniques showed great improvement in treating DDD, hydrogel encapsulation is a laborious process and itself poses several disadvantages such as cell death induced by hydrogel cross-linking, restricted proliferation, and dampened cell–cell interactions. Moreover, upon injection into the injured site, immobilization of cells in the hydrogel matrix limits the cells to properly spread and migrate within the injured site and thus failure to generate new healthy tissues^38^. In addition, hydrogel encapsulation might not be necessary for the induction of chondrogenesis. In one comparative study, simple 3D pellet culture of equine BM-MSC showed the highest degree of chondrogenic differentiation compared with hydrogel, encapsulated systems, in spite of the tendency to generate central core necrosis^37^. We therefore believe that our system might be a better strategy as is takes advantage of natural capacity of 3D cell spheroid in inducing chondrogenesis in vitro without limiting cells’ migration upon treatment into the injury site, in vivo. Gelatin microparticles on the other hand reduce the formation of necrotic cores, and effectively deliver chondrogenic (TGB-β3) and antihypertrophic (MATN3) growth factors for the enhanced differentiation of cells with reduced hypertrophy.

The ultimate goal of cell-based therapy in DDD is to not only provide exogenous, healthy, and chondrogenically active cells, but to also induce chondrogenesis in defective cells in the damaged tissue. Injectable composite ASC spheroids with enhanced chondrogenesis and reduced hypertrophy that secrete chondrogenic cytokines thus offer a promising technique that might address the issues of the currently available therapies for DDD.

## Methods

### Reagents and materials

Gelatin from porcine skin, paraffin oil, SPAN80, EDC, and N-hydroxysuccinimide (NHS) were purchased from Sigma-Aldrich (USA). Glutaraldehyde and acetone were purchased from Daejung Chemicals and Metals (South Korea). TGF-β3 and matrilin-3 were purchased from R&D System (USA). TGF-β3 antibody was purchased from Proteintech (USA) and FITC-labeled matrilin-3 antibody was purchased from Biorbyt (USA). An Alexa Fluor 594 antibody labeling kit was obtained from Life Technologies (USA). StemFit 3D® was purchased from Microfit (South Korea). All culture media were purchased from Hyclone (GE Healthcare, UT, USA). A cell counting kit-8 (CCK-8) assay was purchased from Dojindo (Japan). Trizol reagent was purchased from Qiagen (Hilden, Germany), while the reverse transcription kit and SYBR® Green were purchased from Takara (Japan).

### Fabrication of matrilin-3- and TGF-β3-loaded gelatin microparticles (MATN3–GMP and TGF–β3-GMP)

Matrilin-3 and TGF-β3-loaded GMPs were prepared in accordance with our previous studies via a water-in-oil emulsion method. First, 5 g of gelatin type A (Sigma–Aldrich) bloom 175 was dissolved in 50 mL of distilled deionized water at 40 °C, 700 rpm for 1 h until no visible gelatin particles were present. The gelatin solution was transferred drop-by-drop (1 mL/minute) via a syringe into a flask containing 400 mL of olive oil with 1% SPAN80 for 30 min at RT with an additional 30 min of stirring at 4 °C, 500 rpm to create a water-to-oil emulsion. After 1 h, 40 mM glutaraldehyde was added to the GMP solutions for crosslinking. The stirring was continued for 2 h more. The GMPs were then vacuum-filtered in a Buchner’s funnel and washed with cold acetone. The residual oil was washed by incubating the microparticles in 400 mL of 0.1% Tween-80 solution. The microparticles were finally washed with distilled water, lyophilized, and used for further characterization and experiments.

The proteins matrilin-3 and TGF-β3 were conjugated onto the microparticle via EDC/NHS conjugation. The microparticles were first incubated in an activation buffer containing 50 mM MES hydrate, pH 6.0, for 2 h with constant agitation. They were then collected via centrifugation, counted, and incubated in conjugation buffer (50 mM MES buffer, 4.8 mM EDC, and 48 mM NHS) containing protein solutions at a concentration of 100 ng of matrilin-3 and 100 ng of TGF-β3 per 7500 GMPs at 4 °C overnight. Upon conjugation, the microparticles were washed twice with MES buffer and PBS. Finally, the growth factor-conjugated GMPs were lyophilized and ethylene oxide (EO)-sterilized prior to use.

### Characterization and immunostaining of the GMP microparticles

Dry GMPs were weighed and resuspended in distilled water in preweighted Eppendorf tubes for 30 min and 1 h with constant agitation. Upon swelling, the tubes were spun at 5000 rpm, and all the excess water was removed. The swollen particles were weighed and recorded. The swelling ratio (*q*) was calculated using the equation $q = \frac{{swollen\;MP\;weight\;\left( {W_s} \right)}}{{dry\;MP\;weight\;\left( {W_d} \right)}}$. Finally, the water content was determined with the equation $\left( {\frac{{W_s - W_d}}{{W_s}}} \right) \ast 100.$

For the degradation assay, 5 mg of wet microparticles were resuspended with 10 µg/mL of collagenase I in PBS in Eppendorf tubes. The tubes were spun at specific time points, and 200 µL of the supernatant was collected and replaced with the same volume of new media. The collected samples were then tested for the presence of proteins with the BCA assay, and the measured protein concentration was recorded and plotted against the length of incubation. Microparticles resuspended in PBS served as the negative control.

To determine the presence of conjugated proteins, immunostaining was performed. The primary antibody for TGF-β3 (Proteintech, USA) was first labeled with Alexa Fluor 594 with an antibody-labeling kit in accordance with the manufacturer’s protocol. Conjugated matrilin-3 was visualized using FITC-labeled anti-matrilin-3 antibodies. The labeled antibodies were then added to the GMP solution and incubated for 30 min. The stained GMPs were observed with a fluorescence microscope (CKX41; OLYMPUS, Japan).

### Maintenance of adipose-derived mesenchymal stem cells (ASCs)

With approval from the Institutional Review Board of the Dongguk University Hospital Ethics Committee (IRB no. DUIRB-202006-09), adipose and chondrogenic tissues were obtained via manual isolation from the knee of donor patients from the Dongguk University Hospital, who provided written informed consent. ASCs and DCs were then isolated from the fat and cartilage tissues, respectively, in accordance with the protocol described in our previous study^39^.

ASCs and DCs were maintained in DMEM media (Gibco, BRL) supplemented with 10% FBS, and 1% penicillin/streptomycin (Gibco, BRL). The cells were incubated in humidified air with 5% CO_2_ at 37 °C. The media was changed every other day and the cells were passaged at 80% confluency. ASCs limited to passage 3 were used for all experiments, while DCs were subcultured until passage 6 prior to use. Morphological and molecular characterization of ASCs was done via FACS analysis, while multilineage differentiation potentials were verified via specific staining procedures (Supplementary Figure 2).

### Formation, incorporation of GMP–MATN3/TGF-β3, and chondrogenic differentiation of ASC spheroids

Uniformly sized ASC cell spheroids were generated using StemFit 3D (MicroFIT) cell culture microwells in accordance with the manufacturer’s instructions. Briefly, the microwells were washed with 70% ethanol, washed with 1× PBS twice, and finally coated with an anti-adherent rinsing solution (Stem Cell) for 15 min before use. Trypsin-dissociated ASCs were then seeded into the microwells at a concentration of 5.0 × 10^5^ cells/mL and maintained in low-glucose DMEM media at 37 °C in a 5% CO_2_ incubator for 24 h. The spheroids were then collected and transferred into ultra-low-attachment culture plates and incubated in chondrogenic differentiation media (high‐glucose DMEM medium supplemented with 1% insulin–transferrin–selenium, 50 μg/mL ascorbic acid, and 100 nM dexamethasone without TGF-β1). GMP–MATN3-, GMP–TGFβ3-, or GMP–MIX-incorporated cell spheroids were generated via the same method. A cell suspension containing 5.0 × 10^5^ cells/mL with GMP–MATN3 (100 ng of matrilin-3 per 7500 GMPs), GMP–TGF-β3 (100 ng of TGF-β3 per 7500 GMPs), or GMP–MIX (100 ng of matrilin-3 and 100 ng of TGF-β3 per 7500 GMPs) was seeded into StemFit 3D (MicroFIT) cell culture microwells, incubated for 24 h for spheroid formation, and subsequently transferred to a low-attachment culture plate for chondrogenic differentiation.

Chondrogenic differentiation of ASC spheroids was performed for a total of 14 days, with sampling and analysis for chondrogenesis performed on days 7 and 14. Chondrogenesis and hypertrophy were measured via real-time quantitative PCR, western blot, staining, and immunohistochemical analyses (Supplementary Fig. 1).

### Coculture of ASC spheroids with DCs

At 7 and 14 days post chondrogenic differentiation, the ASC spheroids were cocultured with DCs. Trypsin-dissociated DCs (passage 5) were cultured on a 6-well cell insert at a concentration of 7.5 × 10^4^ cells/well on top of the differentiated spheroids. Both ASC spheroids and DCs were incubated in high‐glucose DMEM medium supplemented with 1% of insulin–transferrin–selenium, 50 μg/mL of ascorbic acid, and 100 nM dexamethasone. Differentiated spheroids served as the source of chondrogenic factors. The coculture was maintained for seven days with media replacement every two days.

### Total RNA extraction and gene expression analysis with qRT-PCR

RNA samples from ASC spheroids containing GMP only, GMP–MATN3, GMP–TGF-β3, and GMP–MIX were extracted and subjected to real-time PCR analysis at days 7 and 14 of differentiation. Total RNA was isolated via the conventional Trizol method (Gibco Invitrogen, Carlsbad, CA). One microgram of the total RNA was then used for cDNA synthesis using TOPscriptTM cDNA synthesis kit (Enzynomics, Daejeon, Korea). PCR analyses were performed with Power Syber Green PCR Master Mix using 1:10 dilutions of the cDNA samples and 10 pmol of the gene-specific primers. The samples were subjected to the following PCR conditions: repeated denaturation at 95 °C for 15 s, annealing at 60 °C for 1 min, and extension at 72 °C for 30 s. The sequences of the primers are listed in Table 1.

**Table 1:** Primers pairs used for real-time quantitative PCR.

Gene	Function	Forward Primer (5′–3′)	Reverse Primer (5′–3′)	Product Size (bp)
SOX9	Chondrogenic Marker	GTA CCC GCA CTT GCA CAA C	TCT CGC TCT CGT TCA GAA GTC	74
COL2A	Chondrogenic Marker	GGG AGT AAT GCA AGG ACC A	ATC ATC ACC AGG CTT TCC AG	175
AGG	Chondrogenic Marker	GCC TGC GCT CCA ATG ACT	ATG GAA CAC GAT GCC TTT CAC	104
COLX	Hypertrophy Marker	ACG CTG AAC GAT ACC AAA TG	TGC TAT ACC TTT ACT CTT TAT GGT GTA	101
MMP13	Hypertrophy Marker	TCA CCA ATT CCT GGG AAG TCT	TCA GGA AAC CAG GTC TGG AG	95
RUNX2	Hypertrophy Marker	CAG ACC AGC AGC ACT CCA TA	CAG CGT CAA CAC CAT CAT TC	178
KI67	Proliferation Marker	CGT CCC AGT GGA AGA GTT GT	CGA CCC CGC TCC TTT TGA TA	143

List of primer pairs for the analysis of chondrogenesis and hypertrophy in ASCs and DCs.

### Protein expression analysis via western blotting

Total protein was extracted from the cells by incubating the ASC spheroids in 1x RIPA lysis buffer. The protein concentrations were quantified using the BCA assay, and 20 ng/µL protein samples were separated via denaturing PAGE gel electrophoresis and transferred to nitrocellulose membranes. The membranes were blocked with 5% nonfat skimmed milk (BD Difco) in TBST solution (Tris-buffered saline Triton ×100) for 1 h and subsequently incubated with primary antibodies (1:500 dilutions for SOX9, ACAN, COL2A, CD44, COLX, and RUNX2) in TBST solution with 5% BSA overnight at 4^o^C. Membranes were then washed, reblocked, and incubated with secondary antibodies (1:2500 dilutions for goat anti-mouse-HRP, goat anti-rabbit-HRP) in TBST solution with 5% nonfat skimmed milk for 1 hr. Detection of immunoreactive bands was performed using E-select (Amersham^TM^, Italy), and images were visualized using Bio-Rad Image Lab software. The antibodies used for western blot analyses are listed in Table 2. All blots derived from the same experiment were processed in parallel.

**Table 2:** Antibodies used for Western Blot analysis.

Antibdoy	Company	Catalog Numbers	Dilution
Rabbit Anti-SOX9	abcam	ab3697	1:500
Mouse Anti-Aggrecan	abcam	ab3778	1:500
Mouse Anti-Collagen Type II	Calbiochem	CP18	1:500
Rabbit Anti-CD 44	abcam	ab157107	1:500
Mouse Anti-Collagen X	Sigma	C7974	1:500
Rabbit Anti-RUNX2	CST	12556 S	1:500
HRP conjugated - Goat Anti-Mouse IgG	cell signaling	7076	1:2500
HRP conjugated - Goat Anti-Rabbit IgG	cell signaling	7074	1:2500

List of antibodies for the analysis of chondrogenesis and hypertrophy in ASCs and DCs.

### Staining and immunohistochemical analysis of differentiated ASC spheroids

Chondrogenic differentiated ASC spheroids were analyzed via Alcian blue staining and immunohistochemistry using conventional protocols. First, cell spheroids were collected and washed with 1 × PBS 2 or 3 times, followed by incubation and fixation in 4% PFA overnight. Fixed-cell spheroids were then embedded in Histogel solution and dehydrated in a series of ethanol solutions with increasing concentrations, cleared in xylene solution, and finally embedded in paraffin. The paraffin blocks were then sectioned using a microtome set for 5 µm per section.

For Alcian blue staining, sections of cell spheroids were rehydrated in ethanol with decreasing concentrations and finally washed with PBS. The tissue sections were incubated in 1% Alcian blue solution (in 3% acetic acid, pH 2.5) for 1 h and were subsequently washed with tap water, dehydrated in a series of increasing ethanol solutions, cleared with xylene, mounted with Acrylamount (StatLab, TX), and visualized under a bright-field microscope.

For collagen-2 immunostaining, sections of cell spheroids were rehydrated in a series of ethanol solutions with decreasing concentrations and finally washed with PBS. The tissue sections were incubated in 5% BSA in PBS blocking solution for 1 h and subsequently incubated in collagen-2 primary antibody (1:500) overnight, washed with 1× PBS, and then further incubated in HRP-conjugated secondary antibody for 2 h. The immunostaining was visualized using liquid DAB + substrate chromogen detection (DAKO). Staining intensities of the spheroid sections were digitally quantified using the ImageJ software.

### Analysis of cytokine secretion

Cytokine secretion in the coculture conditions was analyzed via a Custom Sandwich-based Antibody Array (RayBiotech Inc., Norcross, GA, USA). In this analysis, the media from the coculture setups were collected, centrifuged to remove cells and cell debris, and processed according to the manufacturer’s protocol. Immunoreactivity was detected and visualized using a ChemiDocTM XRS + detection system (BIORAD iNtRON Biotechnology). The signal densities for each protein were semiquantitatively analyzed using Image Lab software (Bio-Rad) and were normalized to the positive controls for each sample.

### In vivo tail IVD degeneration model via nucleus pulposus needle puncture

All animal experiments were performed in accordance with a protocol approved by the Institutional Animal Care and Use Committee (IACUC) of Dongguk University (IACUC-2018-021-2). Briefly, 8-week-old SD rats were raised under specific pathogen-free (SPF) conditions with a light/dark cycle of 12 h, 55–65% humidity, a temperature of 24 ± 3 °C, and free access to food and water. The SD rats were anesthetized by intramuscular injection of xylazine (Rompun, 10 mg/kg; Bayer, Seoul, Korea) with tiletamine hydrochloride/zolazepam hydrochloride (Zoletil, 50 mg/kg; Virbac Laboratories, Carros, France). Tail IVD degeneration was performed by needle puncture after partial subcutaneous incision, as described in previous reports^24,40,41^. In brief, the tail was prepared for aseptic surgery with povidone iodine and alcohol. A 4- to 5-cm subcutaneous incision was performed on Co6/Co7/Co8/Co9 of rat-tail skin. Subsequently, the IVDs of Co6/Co7, Co7/Co8, and Co8/Co9 were punctured by 5 mm via a 21-G spinal needle, and the needle was turned 360° inside the IVD.

A week after DDD induction, each group was injected with 20 μL (5 × 105 cells with GMPs in PBS) in the center of the IVD via a 21-G spinal needle. The SD rats (*n* = 3/3 sites per rat) were divided into five groups (*n* = 9 sites/group) to confirm the synergistic effect of MATN3- and TGF-β3-loaded GMPs in ASC spheroids. After 8 weeks of the initial puncture (seven weeks after implantation), all rats were sacrificed in a CO_2_ gas chamber, and the rat-tail tissues were collected for MRI analysis and tissue staining.

### MRI analysis of IVDs

The MRI analysis was performed as previously described [1]. Briefly, a 4.7-T MRI spectrometer (Bruker Biospec 47/40) and custom MR coil were used for coronal and sagittal T2 MRI with the following settings: time to repetition, 3200 ms; time to echo, 130 ms; matrix, 320 (horizontal) × 320 (vertical); field of view, 120°; and slice thickness, 2 mm, with 0.2-mm spacing between each slice. T2 MRI of all groups was performed to evaluate disc water-content changes 8 weeks after degeneration induction and injection. The T2 signal intensity of the AF and NP of all disks were measured via ImageJ software.

### Statistical analysis

The data are representative of three independent experiments with each experiment done in triplicate. Error bars denote the means ± standard error of mean (SEM) and the differences with p values < 0.05 were considered statistically significant (ns = not significant, *p < 0.05, **p < 0.01, ***p < 0.001; ****p < 0.0001). One-way analysis of variance (ANOVA) followed by Tukey’s post hoc test was used to compare the mean values among groups. Individual data points and *p*-values for significance are indicated in the graph.

### Reporting summary

Further information on research design is available in the Nature Research Reporting Summary linked to this article.

## Supplementary information

Supplementary Information

Reporting Summary

## Data Availability

The authors declare that all data supporting the findings of this study are available within the paper and its supplementary information files. Data are also available from the corresponding author upon reasonable request.
