# Supplementary material for: Matrilin3/TGFβ3 gelatin microparticles promote chondrogenesis, prevent hypertrophy, and induce paracrine release in MSC spheroid for disc regeneration
Source: NPJ Regen Med. 2021 Sep 3;6:50. doi: 10.1038/s41536-021-00160-0 (PMC8417285; doi:10.1038/s41536-021-00160-0)

Matrilin3/TGFβ3 gelatin microparticles promote chondrogenesis, prevent hypertrophy, and induce paracrine release in MSC spheroid for disc regeneration

Alvin Bacero Bello[1,2], Yunkyung Kim[1], Sunghyun Park[3], Manjunatha S Muttigi[1], Jiseong Kim[2], Hansoo Park[1*], and Soohong Lee[2*]

SUPPLEMENTARY FIGURES:

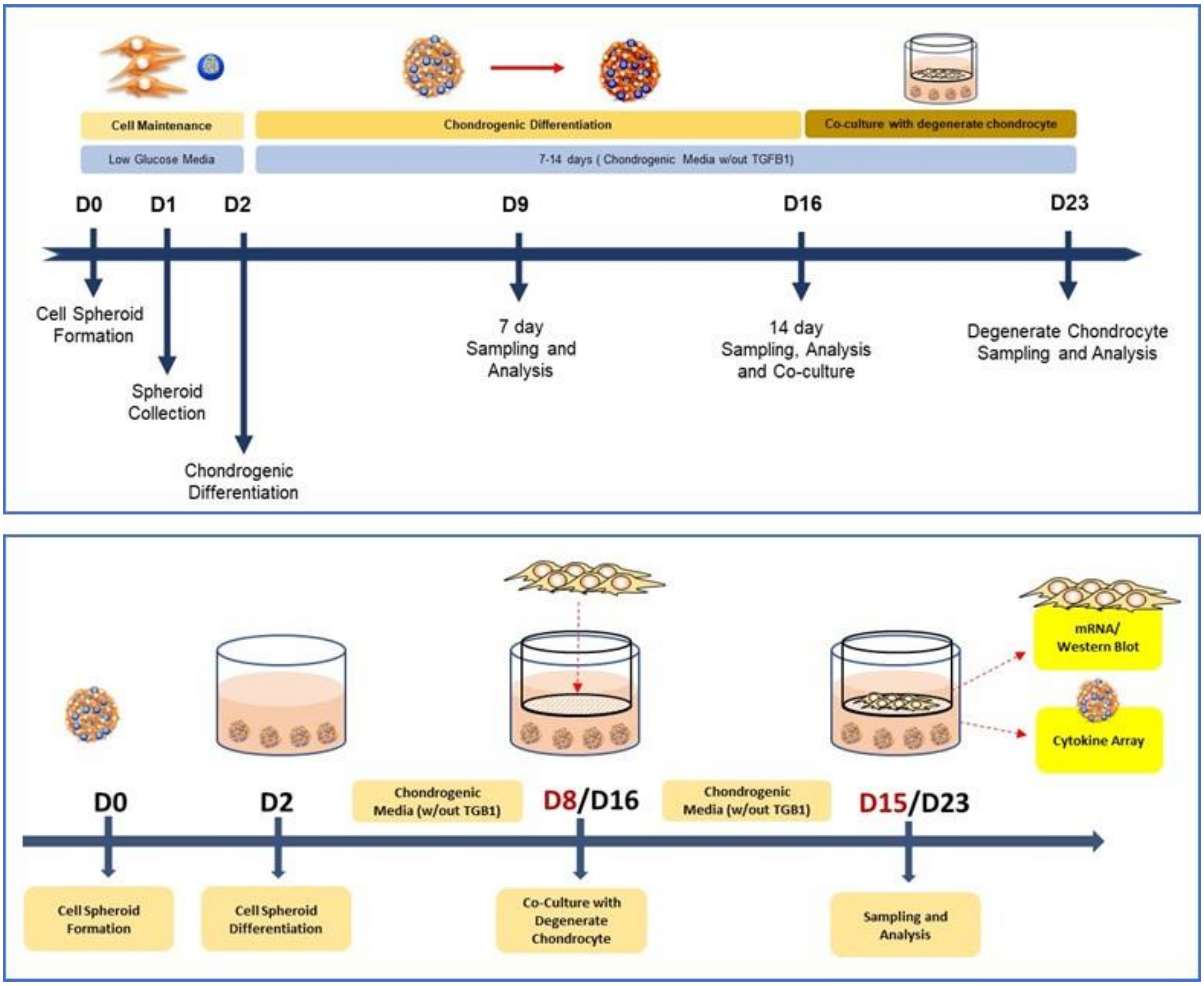

Supplementary Figure 1. Experimental Design: Matrilin-3 andTGFB3 conjugated GMP in MSC chondrogenic differentiation. Growth factors were first conjugated in the GMP, and the resulting GMP composites were incorporated in ASC spheroids and were then differentiated

for 7–14 days in chondrogenic media without TGF-β1. The cell spheroids were then collected and co-cultured with degenerated chondrocytes for another 7 days. After 7 days of co-culture, the degenerated chondrocytes were then collected and subjected to different assays to verify cell regeneration.

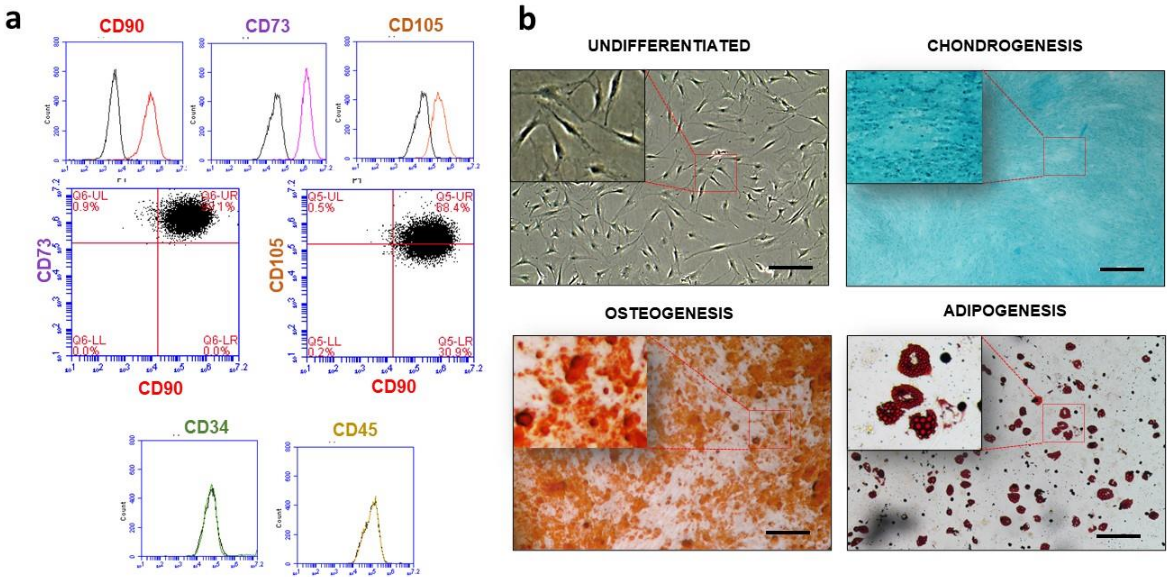

Supplementary Figure 2. Characterization of adipose-derived mesenchymal stem cell (hASCs). Fluorescence-activated cell sorting (FACS) analyses of the MSC surface markers (CD90, CD73, CD105); and MSC-negative markers CD34 and CD45 **(A)**. Representative brightfield images of undifferentiated MSCs at Day 1 (B, upper left), and MSCs undergoing chondrogenic differentiation (B, upper right), osteogenic differentiation (B, lower left), and adipogenic differentiation (B, lower right). Scale bars = 500 μM. Adipogenesis, chondrogenesis, and osteogenesis were conducted for 2, 3, and 4 weeks; respectively.

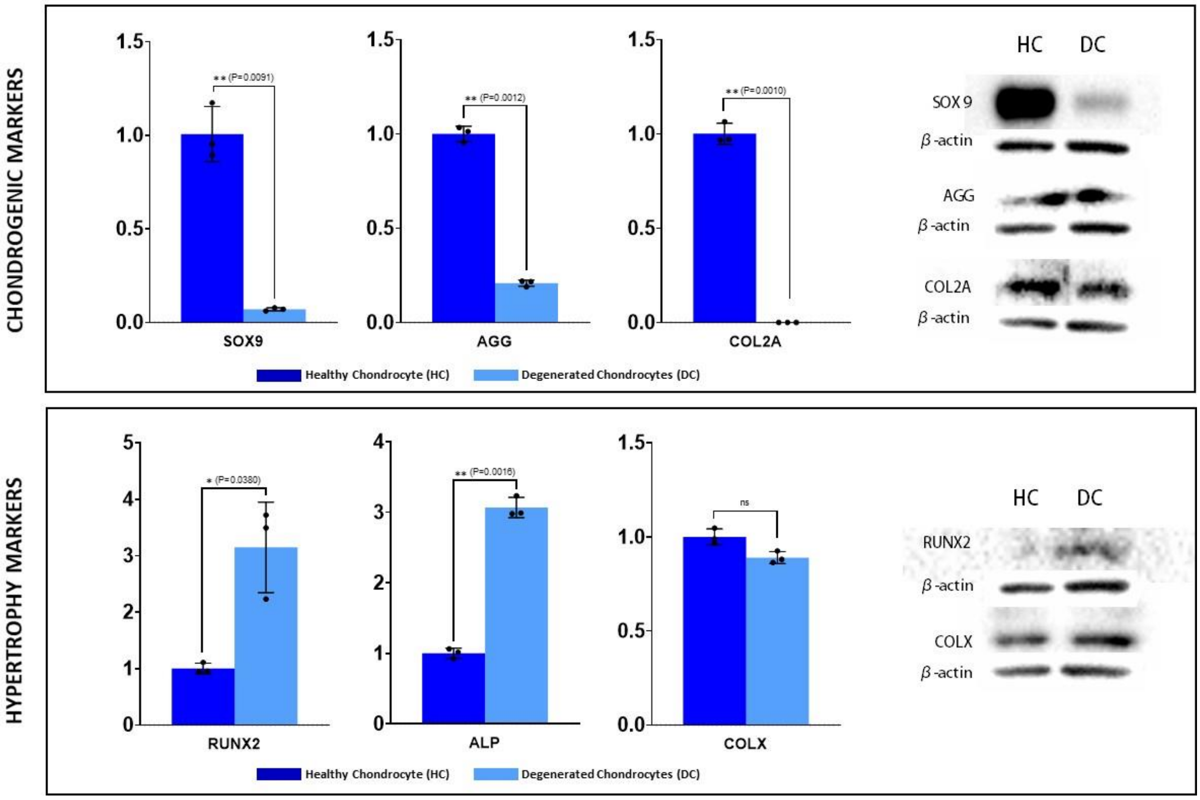

Supplementary Figure 3. Molecular comparisons between healthy chondrocytes (HCs, Passage 0) and degenerated chondrocytes (DCs, Passage 6). Quantitative real-time gene expression analysis and immunoblot analysis of the chondrogenic markers SOX9, AGG, and COL2 (upper panel); and hypertrophy markers COLX, RUNX2, and ALP (upper panel). RT-PCR data were normalized against GAPDH expression. The data are representative of three independent experiments in triplicate. Error bars denote the means ± s.d. (ns = not significant, * $p < 0.05$, ** $p < 0.01$, *** $p < 0.001$; ****, $p < 0.0001$). Individual data points and p-values for significance are indicated in the graph.

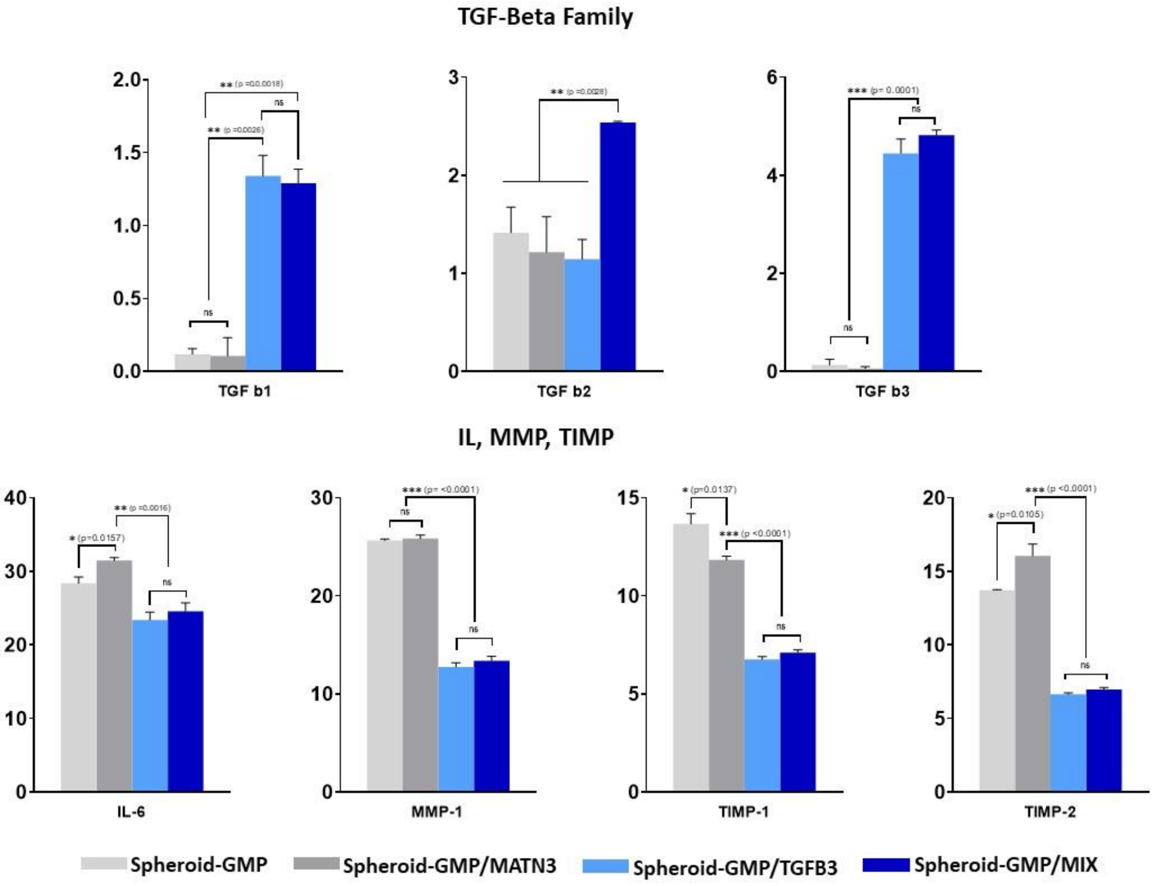

Supplementary Figure 4. Upregulated/downregulated cytokines secreted by day 7 spheroids. Immunoblot analysis (cytokine array format) of the various cytokines (TGF-β1, TGF-β2, TGF-β3, IL6, MMP-1, TIMP1, TIMP2) secreted by Day 7 chondrogenic spheroids. Values were normalized against positive controls. The data are representative of three independent experiments in triplicate. Error bars denote the means ± s.d. (ns = not significant, * $p < 0.05$, ** $p < 0.01$, *** $p < 0.001$; ****, $p < 0.0001$). Individual data points and p-values for significance are indicated in the graph.

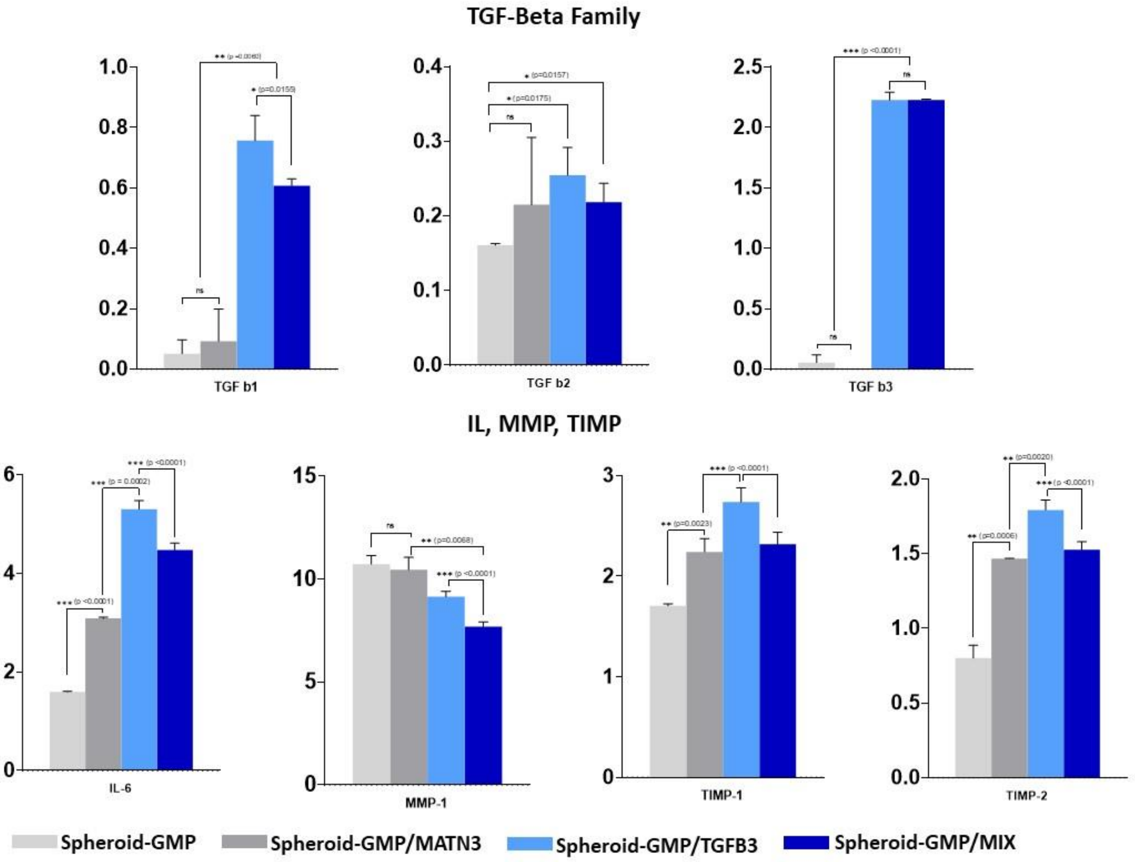

Supplementary Figure 5. Upregulated/Downregulated cytokines secreted by Day 14 spheroids. Immunoblot analysis (cytokine array format) of the various cytokines (TGF-β1, TGF-β2, TGF-β3, IL6, MMP-1, TIMP1, TIMP2) secreted by Day 7 chondrogenic spheroids. Values were normalized against positive control. The data are representative of three independent experiments in triplicate. Error bars denote the means ± s.d. (ns = not significant, * $p < 0.05$, ** $p < 0.01$, *** $p < 0.001$; ****, $p < 0.0001$). Individual data points and p-values for significance are indicated in the graph.

DAY 7 CHONDROGENIC MARKERS

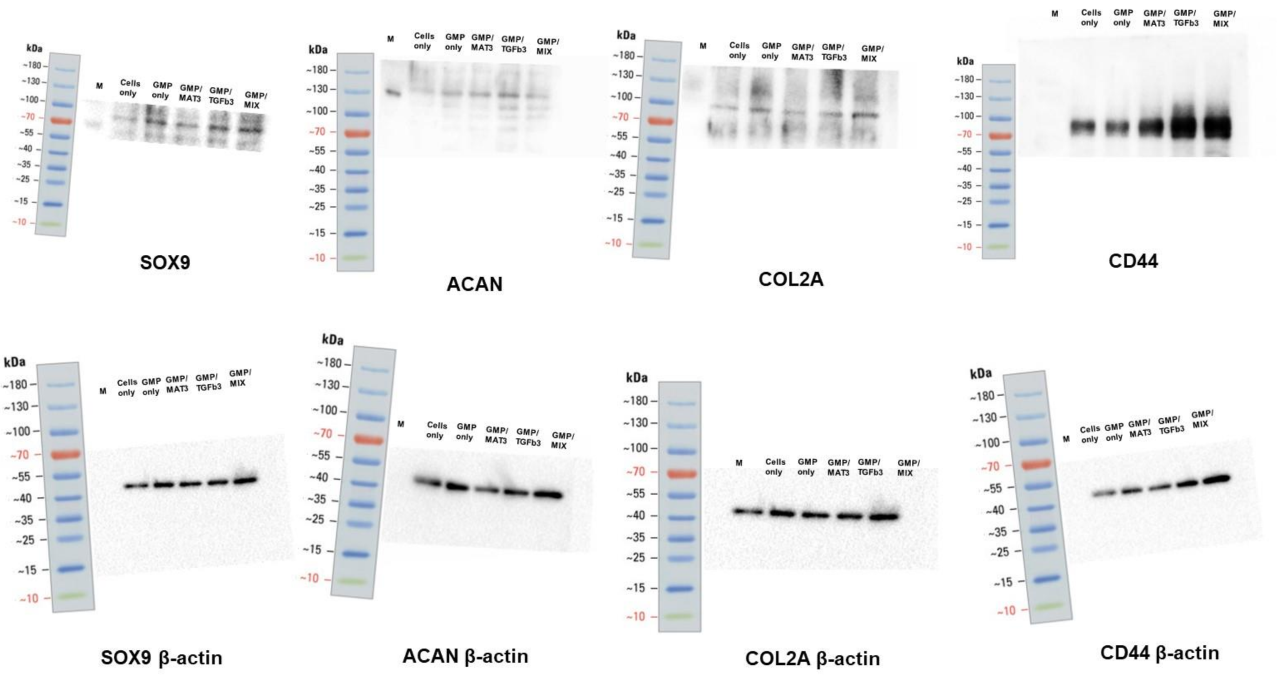

DAY 14 CHONDROGENIC MARKERS

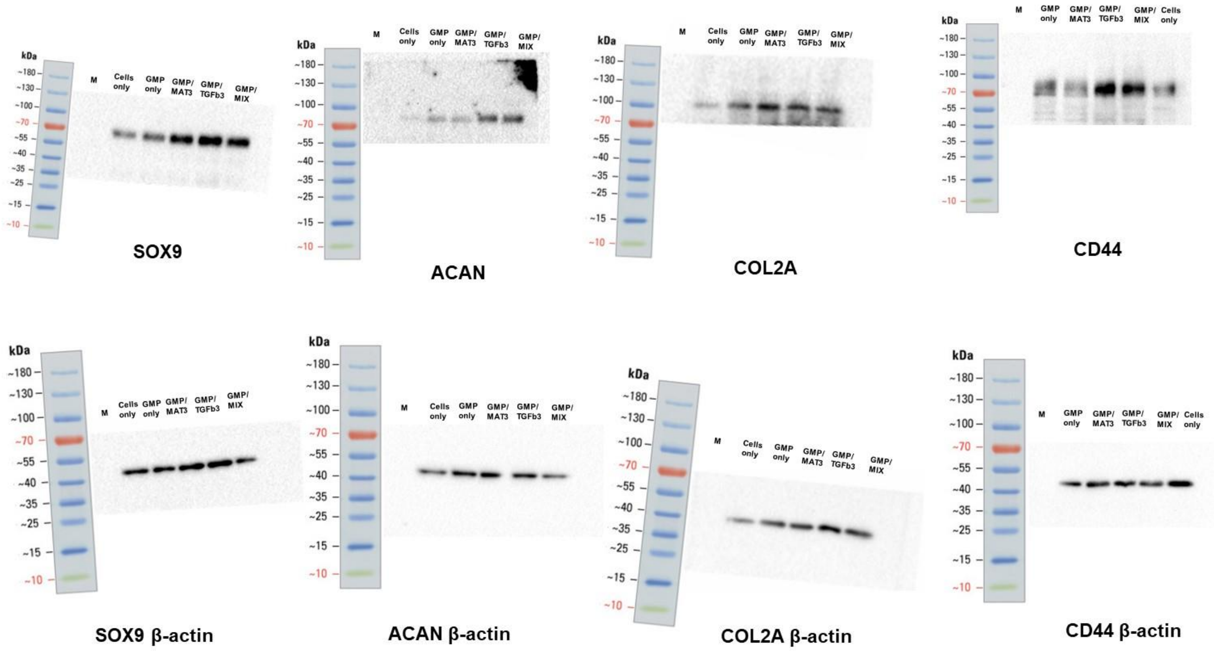

DAY 7 HYPERTROPHY MARKERS

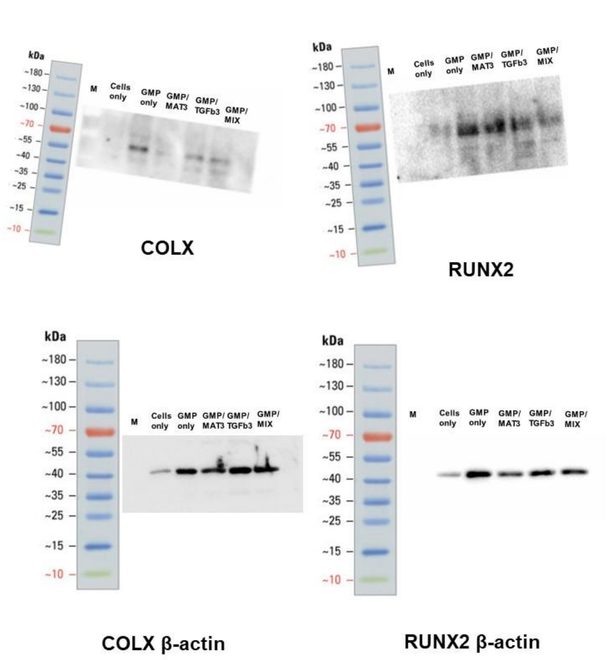

DAY 14 HYPERTROPHY MARKERS

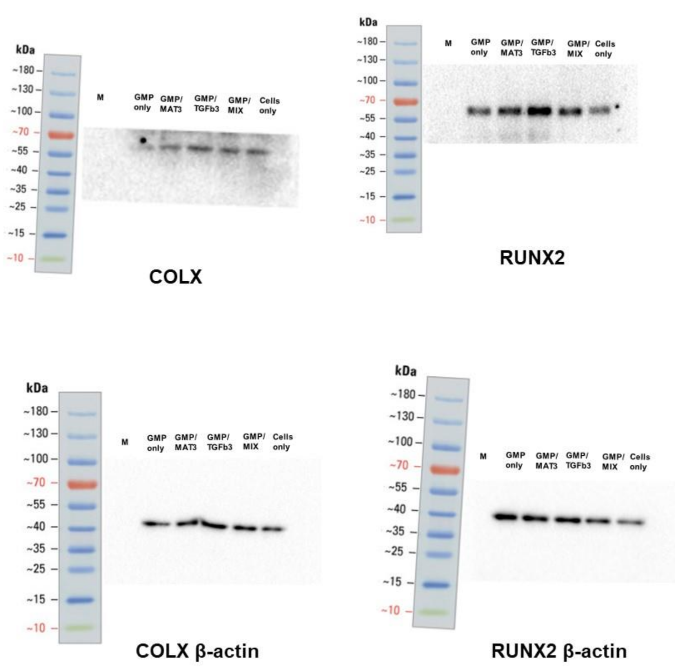

Supplement: Supplementary file 1 — Supplementary Information [file 41536_2021_160_MOESM1_ESM.pdf]
