# Supplementary material for: Matrilin3/TGFβ3 gelatin microparticles promote chondrogenesis, prevent hypertrophy, and induce paracrine release in MSC spheroid for disc regeneration
Source: NPJ Regen Med. 2021 Sep 3;6:50. doi: 10.1038/s41536-021-00160-0 (PMC8417285; doi:10.1038/s41536-021-00160-0)
Supplement: Supplementary file 2 — Reporting Summary [file 41536_2021_160_MOESM2_ESM.pdf]

nature research

Corresponding author(s): Soohong Lee and Hansoo Park

Last updated by author(s): Jul 9, 2021

Reporting Summary

Nature Research wishes to improve the reproducibility of the work that we publish. This form provides structure for consistency and transparency in reporting. For further information on Nature Research policies, see our Editorial Policies and the Editorial Policy Checklist.

Statistics

For all statistical analyses, confirm that the following items are present in the figure legend, table legend, main text, or Methods section.

n/a	Confirmed	
☐	☒	The exact sample size (*n*) for each experimental group/condition, given as a discrete number and unit of measurement
☐	☒	A statement on whether measurements were taken from distinct samples or whether the same sample was measured repeatedly
☐	☒	The statistical test(s) used AND whether they are one- or two-sided<br>*Only common tests should be described solely by name; describe more complex techniques in the Methods section.*
☒	☐	A description of all covariates tested
☒	☐	A description of any assumptions or corrections, such as tests of normality and adjustment for multiple comparisons
☐	☒	A full description of the statistical parameters including central tendency (e.g. means) or other basic estimates (e.g. regression coefficient) AND variation (e.g. standard deviation) or associated estimates of uncertainty (e.g. confidence intervals)
☐	☒	For null hypothesis testing, the test statistic (e.g. *F*, *t*, *r*) with confidence intervals, effect sizes, degrees of freedom and *P* value noted<br>*Give P values as exact values whenever suitable.*
☒	☐	For Bayesian analysis, information on the choice of priors and Markov chain Monte Carlo settings
☒	☐	For hierarchical and complex designs, identification of the appropriate level for tests and full reporting of outcomes
☒	☐	Estimates of effect sizes (e.g. Cohen's *d*, Pearson's *r*), indicating how they were calculated

Our web collection on statistics for biologists contains articles on many of the points above.

Software and code

Policy information about availability of computer code

Data collection	No software was used for data collection.
Data analysis	For statistical data analysis, Graphpad Prism 9 was used.

For manuscripts utilizing custom algorithms or software that are central to the research but not yet described in published literature, software must be made available to editors and reviewers. We strongly encourage code deposition in a community repository (e.g. GitHub). See the Nature Research guidelines for submitting code & software for further information.

Data

Policy information about availability of data

All manuscripts must include a data availability statement. This statement should provide the following information, where applicable:

- Accession codes, unique identifiers, or web links for publicly available datasets
- A list of figures that have associated raw data
- A description of any restrictions on data availability

The authors declare that all data supporting the findings of this study are available within the paper and its supplementary information files.

Field-specific reporting

Please select the one below that is the best fit for your research. If you are not sure, read the appropriate sections before making your selection.

☒ Life sciences ☐ Behavioural & social sciences ☐ Ecological, evolutionary & environmental sciences

For a reference copy of the document with all sections, see nature.com/documents/nr-reporting-summary-flat.pdf

Life sciences study design

All studies must disclose on these points even when the disclosure is negative.

Sample size	All experiments were done in three independent trials, each in triplicate (n=9) to ensure statistical validity.
Data exclusions	No data were excluded from the analyses.
Replication	All experiments involve three independent experiments done in triplicates.
Randomization	Rats for the in-vivo study were randomly selected for the control and treatment groups.
Blinding	Investigators were blinded in sample allocation for data collection and analyses.

Reporting for specific materials, systems and methods

We require information from authors about some types of materials, experimental systems and methods used in many studies. Here, indicate whether each material, system or method listed is relevant to your study. If you are not sure if a list item applies to your research, read the appropriate section before selecting a response.

Materials & experimental systems

n/a	Involved in the study	
☐	☒	Antibodies
☒	☐	Eukaryotic cell lines
☒	☐	Palaeontology and archaeology
☐	☒	Animals and other organisms
☒	☐	Human research participants
☒	☐	Clinical data
☒	☐	Dual use research of concern

Methods

n/a	Involved in the study	
☒	☐	ChIP-seq
☒	☐	Flow cytometry
☒	☐	MRI-based neuroimaging

Antibodies

Antibodies used

Rabbit Anti-TGFB3 (Biorbyt, Cat#: orb214663)
Rabbit Anti-Matrilin 3 (Biorbyt, Cat#: orb30989)
Rabbit Anti-SOX9 (abcam, Cat#:ab3697)
Mouse Anti-Aggrecan,(abcam, Cat#:ab3778)
Mouse Anti-Collagen Type II (Calbiochem, Cat#:CP18)
Rabbit Anti-CD 44 (abcam, Cat#:ab157107)
Mouse Anti-Collagen X (Sigma, Cat#:C7974)
Rabbit Anti-RUNX2 (CST, Cat#:12556S)
HRP conjugated - Goat Anti-Mouse IgG (cell signaling, Cat#:7076)
HRP conjugated - Goat Anti-Rabbit IgG (cell signaling, Cat#:7074)

Validation

All antibodies were validated via Western Blot Analysis

Animals and other organisms

Policy information about studies involving animals; ARRIVE guidelines recommended for reporting animal research

Laboratory animals	8-week-old male Sprague-Dawley (SD) rats (Orient-bio, Seongnam, Korea).
Wild animals	This study did not involve any wild animals.
Field-collected samples	This study did not involve any field-collected samples.

Ethics oversight	All animal experiments were performed in accordance with a protocol approved by the Institutional Animal Care and Use Committee (IACUC) of Dongguk university (IACUC-2018-021-2)

Note that full information on the approval of the study protocol must also be provided in the manuscript.
